# Supplementary figures and images for: The mTORC2 subunit RICTOR drives breast cancer progression by promoting ganglioside biosynthesis through transcriptional and epigenetic mechanisms
Source: PLoS Biol. 2025 Sep 11;23(9):e3003362. doi: 10.1371/journal.pbio.3003362 (PMC12425323; doi:10.1371/journal.pbio.3003362)

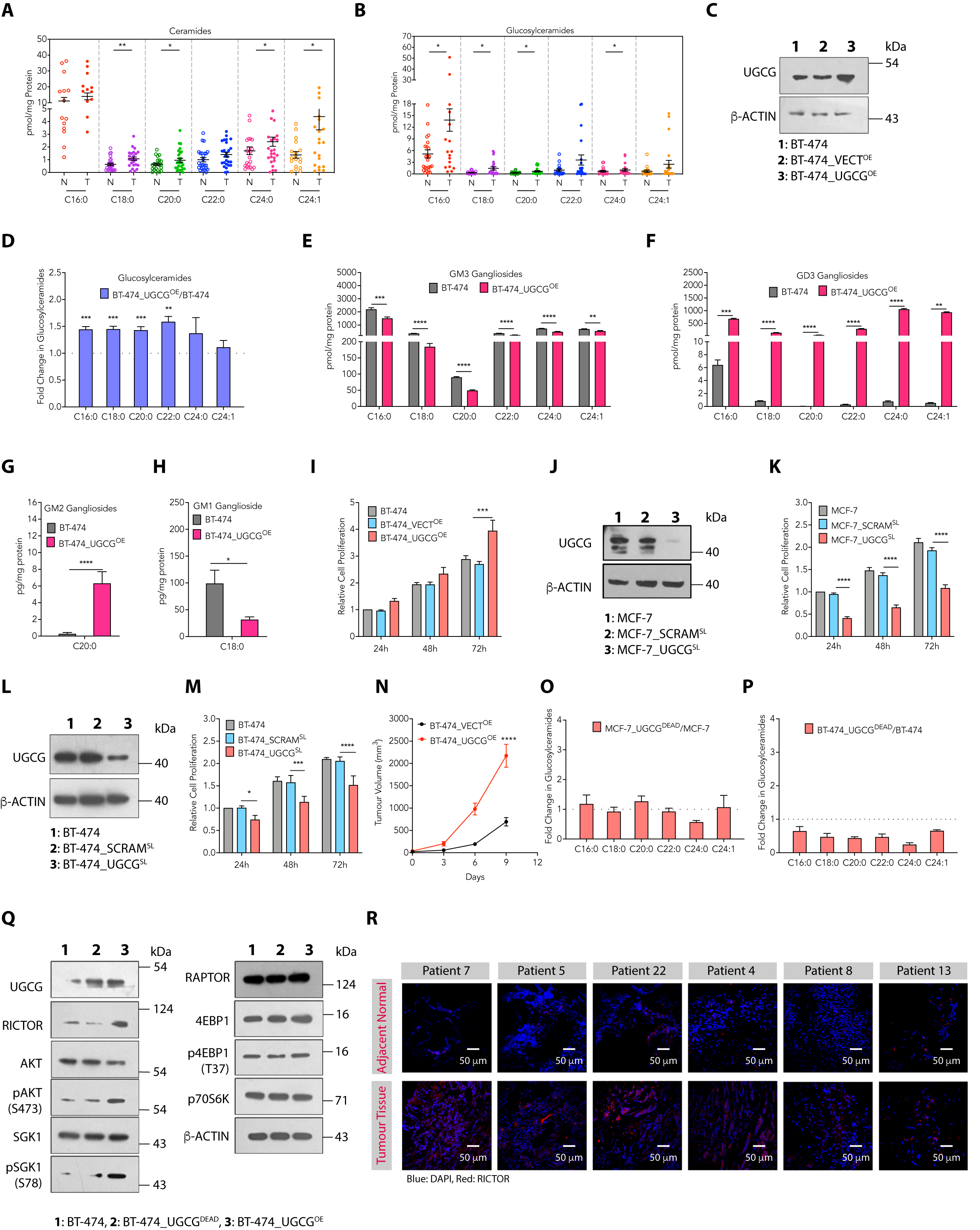

Supplement: S1 Fig — (A, B) Absolute quantitation (pmol/mg protein) (mean ± SEM, n = 27) of different species of ceramides (A) and glucosylceramides (B) shows higher levels in luminal tumor tissues (labeled as T) in comparison to adjacent normal tissues (labeled as N). (C) Immunoblot confirm increased UGCG expression in BT-474_UGCGOE cells compared to BT-474 cells. (D) Quantification of glucosylceramides (mean ± SEM, n = 4) confirms an increase in BT-474_UGCGOE cells over BT-474 cells. (E–H) Absolute quantification (mean ± SEM, n = 3–4) of GM3 (E), GD3 (F), GM2 (G), and GM1 (H) gangliosides shows an increase in GD3 and GM2 gangliosides and a decrease in GM3 and GM1 gangliosides in BT-474_UGCGOE cells compared to BT-474 cells. (I) Cell proliferation (mean ± SEM, n = 5) assay demonstrates an increase in the proliferation of BT-474_UGCGOE cells over BT-474_VECTOE cells. (J–M) Immunoblot confirms silencing of UGCG in MCF-7 and BT-474 cells (J, L) and cell proliferation assay demonstrates a decrease in proliferation in MCF-7_UGCGSL and BT-474_UGCGSL cells over MCF-7_SCRAMSL (mean ± SEM, n = 5) and BT-474_SCRAMSL cells (mean ± SEM, n = 3) (K, M). (N) Tumor growth kinetics reveal enhanced growth of BT-474_UGCGOE tumors compared to BT-474_VECTOE tumors (mean ± SEM, n = 5). (O, P) Fold change of glucosylceramides (mean ± SEM, n = 4) confirms no significant increase in glucosylceramides in MCF-7_UGCGDEAD and BT-474_UGCGDEAD cells compared to MCF-7 and BT-474 cells. (Q) Immunoblots show the expression of RICTOR, RAPTOR, AKT, pAKTSer473, SGK1, pSGK1Ser78, 4EBP1, p4EBP1Thr37 and p70S6K in BT-474_UGCGOE cells in comparison to BT-474_UGCGDEAD cells. (R) Immunofluorescence images show elevated RICTOR expression in tumor tissues compared to adjacent normal tissue sections. Data among groups were analyzed using a paired Student t test (for patient data), One-way ANOVA among multiple groups or Two-way ANOVA in time-dependent studies. P-value: *p < 0.05, **p < 0.01, ***p < 0.0005, ****p < 0.0001. Numerical [file pbio.3003362.s001.tif]

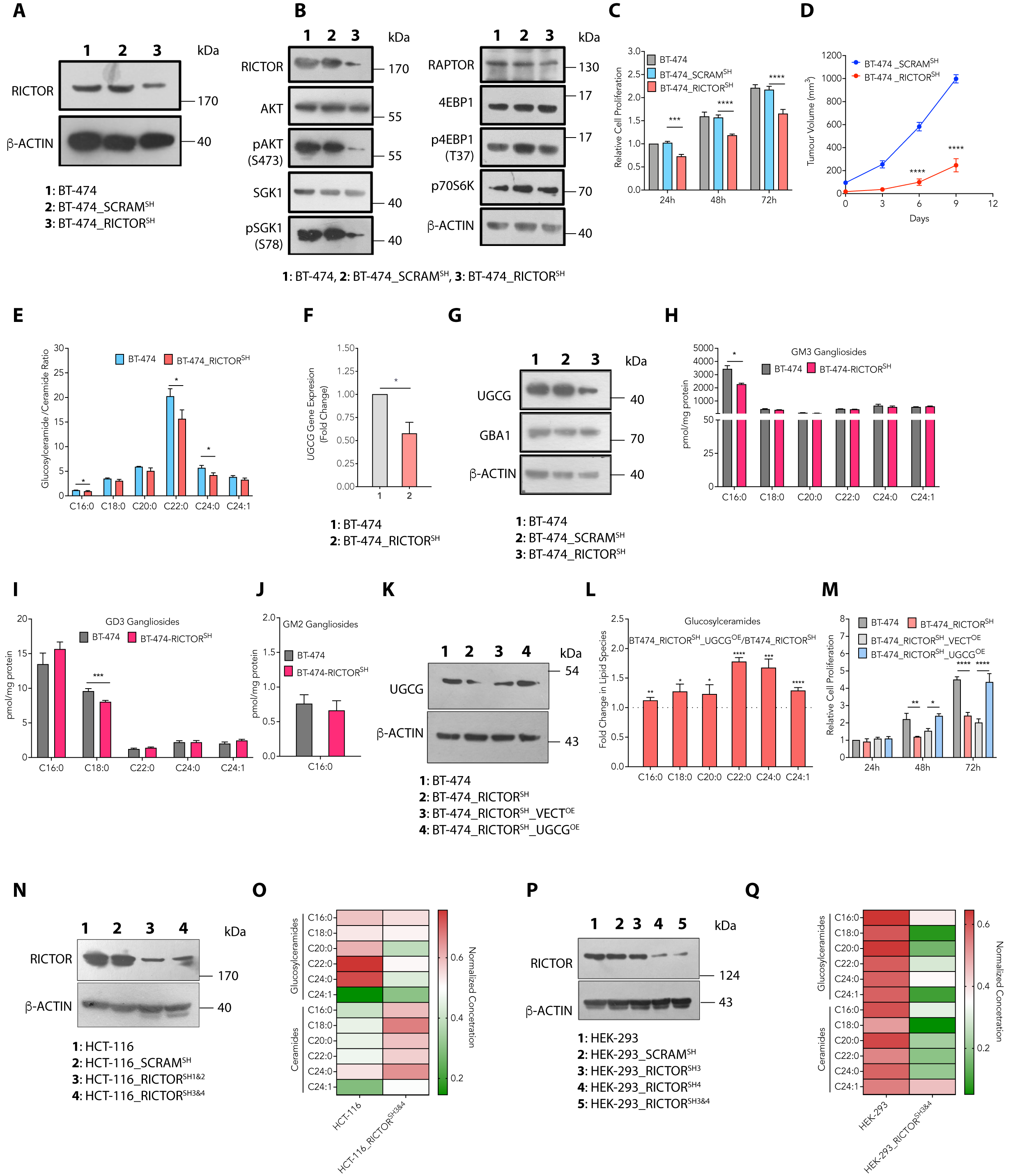

Supplement: S2 Fig — (A) Immunoblots confirm knockdown of RICTOR expression in BT-474_RICTORSH cells. (B) Immunoblots show changes in expression of RICTOR, RAPTOR, and their downstream effectors in BT-474_RICTORSH cells compared to BT-474_SCRAMSH cells. (C) Cell proliferation studies show a decrease in the proliferation of BT-474_RICTORSH cells (mean ± SEM, n = 3) compared to BT-474_SCRAMSH cells. (D) Tumor growth kinetics show significantly slower growth of BT-474–7_RICTORSH (mean ± SEM, n = 5) tumors compared to BT-474_SCRAMSH tumors. (E) Altered glucosylceramide/ceramide ratio reveals a decrease in glucosylceramides in BT-474_RICTORSH cells compared to BT-474 cells. (F, G) qRT-PCR (mean ± SEM, n = 4) (F) and immunoblots (G) demonstrate downregulation of UGCG without any change in GBA1 expression in BT-474_RICTORSH cells compared to BT-474_SCRAMSH cells. (H–J) Absolute quantification (mean ± SEM, n = 3–5) of GM3 (H), GD3 (I), and GM2 (J) gangliosides shows a decrease in GM3 and GD3 gangliosides in BT-474_RICTORSH cells compared to BT-474 cells. (K) Immunoblot shows the confirmation of UGCG overexpression in BT-474_RICTORSH cells. (L) Fold changes in glucosylceramides (mean ± SEM, n = 4) in BT-474_RICTORSH_UGCGOE cells compared to BT-474_RICTORSH cells confirm an increase in glucosylceramides. (M) Cell proliferation assay demonstrates an increase in cell proliferation (mean ± SEM, n = 3) of BT-474_RICTORSH cells on overexpression of UGCG. (N–Q) Immunoblot showing the confirmation of RICTOR knockdown in HCT-116_RICTORSH (N) and HEK-293_RICTORSH cells (P), and quantification of glucosylceramides in HCT-116_RICTORSH (O) and HEK-293_RICTORSH (Q) cells compared to HCT-116 and HEK-293 cells. Data among groups were analyzed using an unpaired Student t test or One-way ANOVA among multiple groups or Two-way ANOVA in time-dependent studies. p-value: **p < 0.01, ***p < 0.001, ****p < 0.0001. Numerical data can be found in S2 Dataset. (S2_Fig.TIF) [file pbio.3003362.s002.tif]

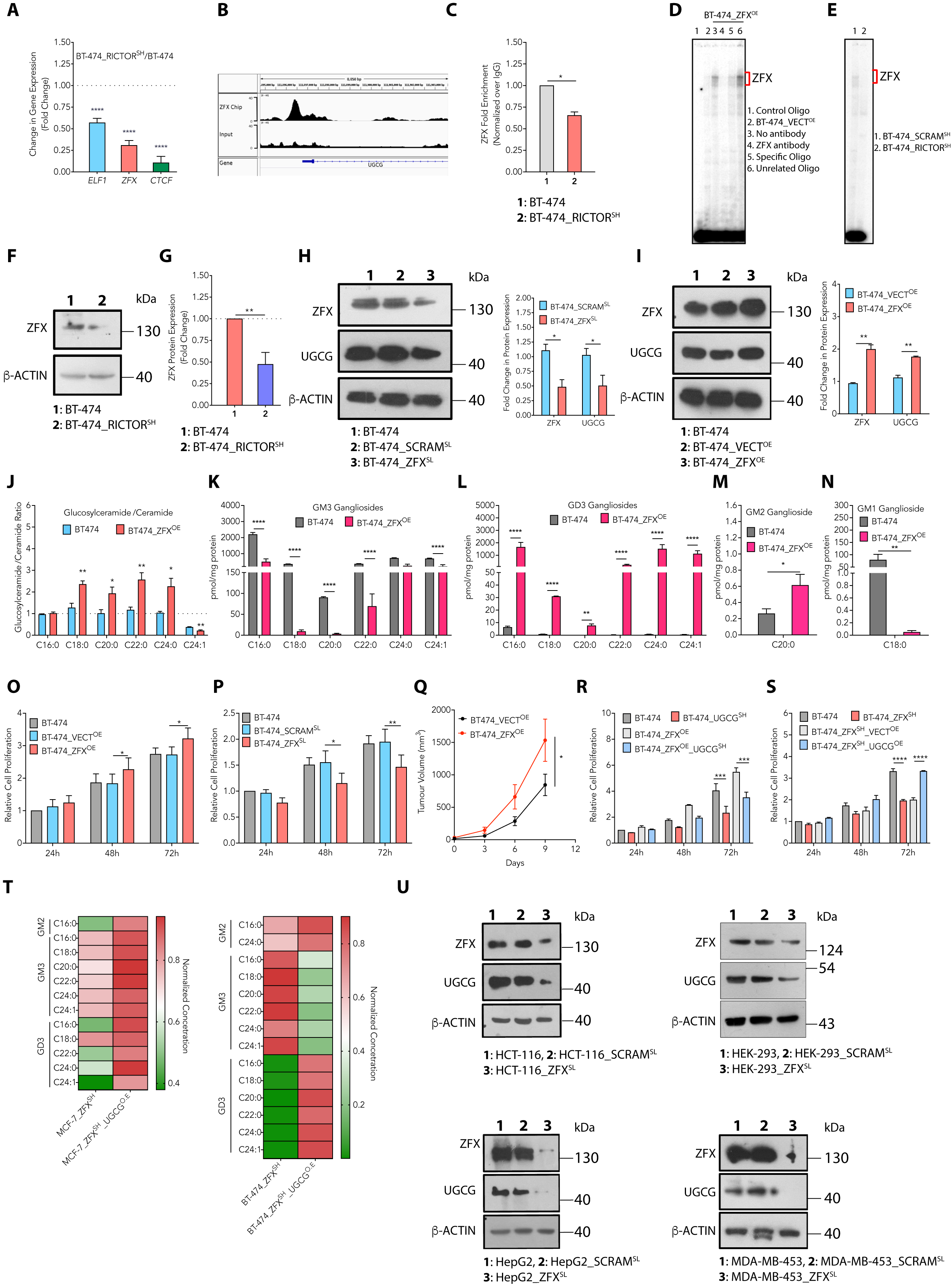

Supplement: S3 Fig — (A) qRT-PCR (mean ± SEM, n = 4) confirms reduced expression of RICTOR-regulated ELF1, ZFX, and CTCF transcription factors in BT-474_RICTORSH cells. (B) University of California, Santa Cruz browser view of ZFX peaks on the promoter region of the UGCG gene as determined by ChIP-seq analysis. (C) ChIP-qPCR (mean ± SEM, n = 3) results show reduced binding of ZFX to the UGCG promoter in BT-474_RICTORSH cells. (D) EMSA shows the binding of ZFX to the UGCG promoter (lanes 2 and 3) in BT-474–7_ZFXOE cells, shift-ablation assay in BT-474_ZFXOE cells (lane 4), competition assay with specific (lane 5) and unrelated oligo as a control (lane 6). (E) EMSA comparing endogenous ZFX-DNA binding activity between BT-474_SCRAMSH and BT-474_RICTORSH cells. (F, G) Immunoblot and its quantification (mean ± SEM, n = 3) show downregulation of ZFX expression in BT-474_RICTORSH cells. (H, I) Immunoblots and their quantification (mean ± SEM, n = 3) confirm that silencing of ZFX decreases UGCG expression (H) and overexpression of ZFX enhances UGCG expression in BT-474 cells (I). (J) The glucosylceramide to ceramide ratio confirms an increase in glucosylceramides in BT-474–7_ZFXOE cells. (K–N) Absolute quantification (mean ± SEM, n = 3–4) of GM3 (K), GD3 (L), GM2 (M), and GM1 (N) gangliosides shows an increase of GD3 and GM2 gangliosides and a decrease of GM3 and GM1 gangliosides in BT-474_ZFXOE cells. (O, P) Cell proliferation assay confirms an increase in cell proliferation of BT-474_ZFXOE cells (mean ± SEM, n = 5) (O), whereas BT-474_ZFXSL cells (mean ± SEM, n = 3) show decreased cell proliferation (P). (Q) Tumor growth kinetics recorded a significantly higher growth of BT-474_ZFXOE (mean ± SEM, n = 5) than BT-474_VECTOE tumors. (R, S) Cell proliferation demonstrates a decrease in proliferation of BT-474_ZFXOE cells on UGCG silencing (mean ± SEM, n = 4) (R), whereas BT-474_ZFXSH cells (mean ± SEM, n = 3) show enhanced cell proliferation on UGCG overexpression (S). (T) Changes in gangliosides [file pbio.3003362.s003.tif]

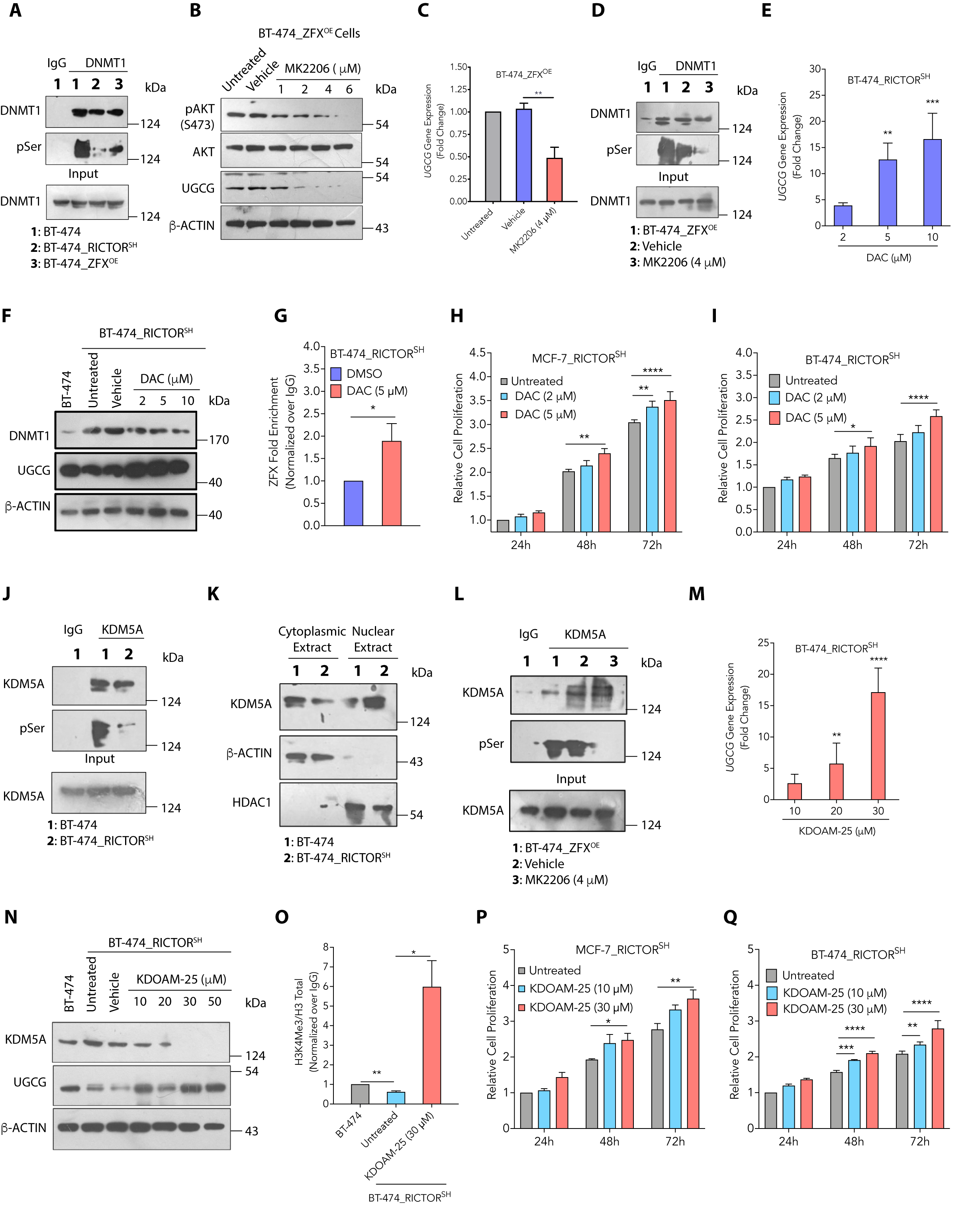

Supplement: S4 Fig — (A) Immunoblot showing alteration in phosphorylation of DNMT1 by pan-phospho-ser antibody in BT-474_RICTORSH and BT-474_ZFXOE cells compared to BT-474 cells. (B) Immunoblots showing a dose-dependent decrease in pAKT and UGCG expression in BT-474_ZFXOE cells on treatment with AKT inhibitor MK2206. (C) Results from qRT-PCR (mean ± SEM, n = 4) show a decrease in UGCG expression in BT-474_ZFXOE cells on AKT inhibition by MK2206 (4 μM). (D) Immunoblot reveals a decrease in phosphorylation of DNMT1 by pan-phospho-ser antibody on treatment of BT-474_ZFXOE cells with AKT inhibitor MK2206. (E, F) Results from qRT-PCR (mean ± SEM, n = 4) (E) and immunoblot (F) show increased UGCG expression in BT-474_RICTORSH cells on DNMT inhibition by DAC. (G) ChIP-qPCR results (mean ± SEM, n = 3) confirm enhanced binding of ZFX to UGCG promoter in BT-474_RICTORSH cells on DAC (5 μM) treatment. (H, I) Cell proliferation confirms increased proliferation of MCF-7_RICTORSH (mean ± SEM, n = 3) (H) and BT-474_RICTORSH (mean ± SD, n = 3) cells (I) on DAC treatment. (J) Immunoblot showing the change in phosphorylation of KDM5A using the pan-phospho-Ser antibody in BT-474_RICTORSH cells compared to BT-474 cells. (K) Immunoblot shows alterations in the levels of KDM5A in nuclear and cytoplasmic extracts in BT-474_RICTORSH cells compared to BT-474 cells. (L) Immunoblot reveals a decrease in phosphorylation of KDM5A on treatment of BT-474_ZFXOE cells with AKT inhibitor MK2206. (M, N) Results from qRT-PCR (mean ± SEM, n = 4) (M) and immunoblot (N) show increased UGCG expression in BT-474_RICTORSH on KDM5A inhibition. (O) ChIP-qPCR results (mean ± SEM, n = 3) show a reduced H3K4Me3 mark on UGCG promoter in BT-474_RICTORSH cells that increases on treatment with KDOAM-25 inhibitor (30 μM). (P, Q) Cell proliferation assay confirms increased proliferation of MCF-7_RICTORSH (mean ± SEM, n = 3) (P) and BT-474_RICTORSH cells (mean ± SEM, n = 3) (Q) on KDM5A inhibition. Data among the two groups were analyzed u [file pbio.3003362.s004.tif]

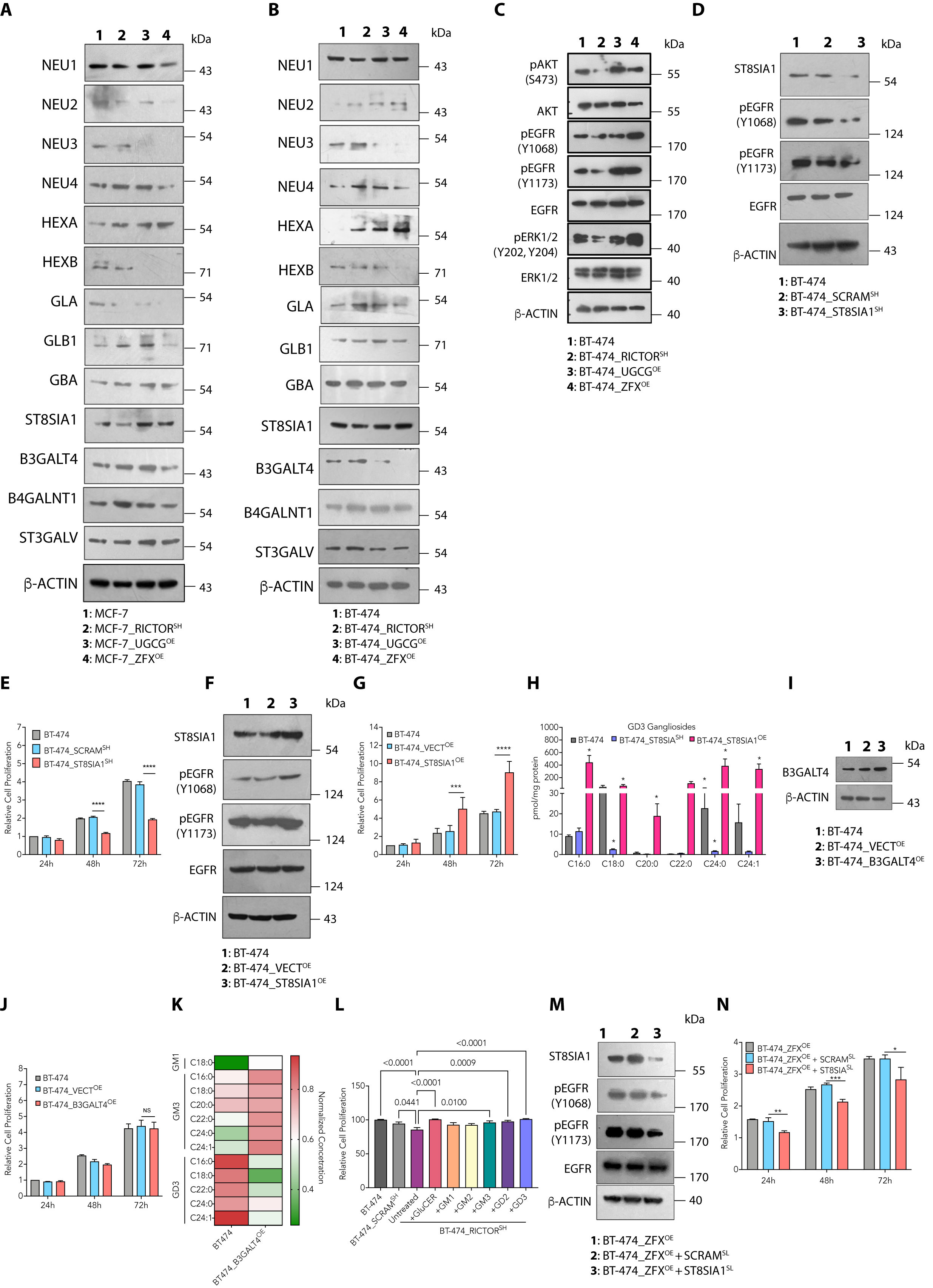

Supplement: S5 Fig — (A, B) Immunoblots showing changes in the expression of different enzymes of the ganglioside metabolic pathway in MCF-7_RICTORSH, MCF-7_UGCGOE, and MCF-7_ZFXOE cells compared to MCF-7 cells (A) and in BT-474_RICTORSH, BT-474_UGCGOE, and BT-474_ZFXOE cells compared to BT-474 cells (B). (C) Immunoblots reveal elevated pEGFRY1173 and pEGFRY1068 levels in BT-474_UGCGOE and BT-474–7_ZFXOE cells compared to BT-474 cells. (D) Immunoblots show attenuated EGFR activation on shRNA-mediated silencing of GD3 synthase (ST8SIA1) in BT-474 cells. (E) Cell proliferation assay demonstrates a decrease in cell proliferation (mean ± SEM, n = 3) of BT-474_ST8SIA1SH cells compared to BT-474_SCRAMSH cells. (F) Immunoblots show enhanced EGFR activation on overexpression of ST8SIA1 in BT-474 cells. (G) Cell proliferation assay demonstrates increased proliferation (mean ± SEM, n = 3) of BT-474_ST8SIA1OE cells compared to BT-474_VECTOE cells. (H) Absolute quantification (mean ± SEM, n = 3) of GD3 gangliosides validates the silencing and overexpression of ST8SIA1 in BT-474 cells. (I) Immunoblots confirm overexpression of B3GALT4 in BT-474 cells. (J) Cell proliferation assay (mean ± SEM, n = 4) of BT-474_B3GALT4OE cells. (K) Absolute quantification (mean ± SEM, n = 4–5) of gangliosides validates the overexpression of B3GALT4 in BT-474 cells with an increase in GM1 gangliosides and a decrease in GD3 gangliosides. (L) Cell proliferation assay (mean ± SEM, n = 3) showing an increase in proliferation of BT-474_RICTORSH cells upon supplementing GD3 gangliosides. (M) Immunoblots show attenuated EGFR activation on siRNA-mediated silencing of ST8SIA1 in BT-474_ZFXOE cells. (N) Cell proliferation assay (mean ± SEM, n = 3) demonstrates a decrease in cell proliferation of BT-474_ZFXOE cells on siRNA-mediated inhibition of ST8SIA1. Data among two groups were analyzed using an unpaired Student t test, among multiple groups using One-way ANOVA, and by Two-way ANOVA in time-dependent studies. p-value: *p < 0. [file pbio.3003362.s005.tif]

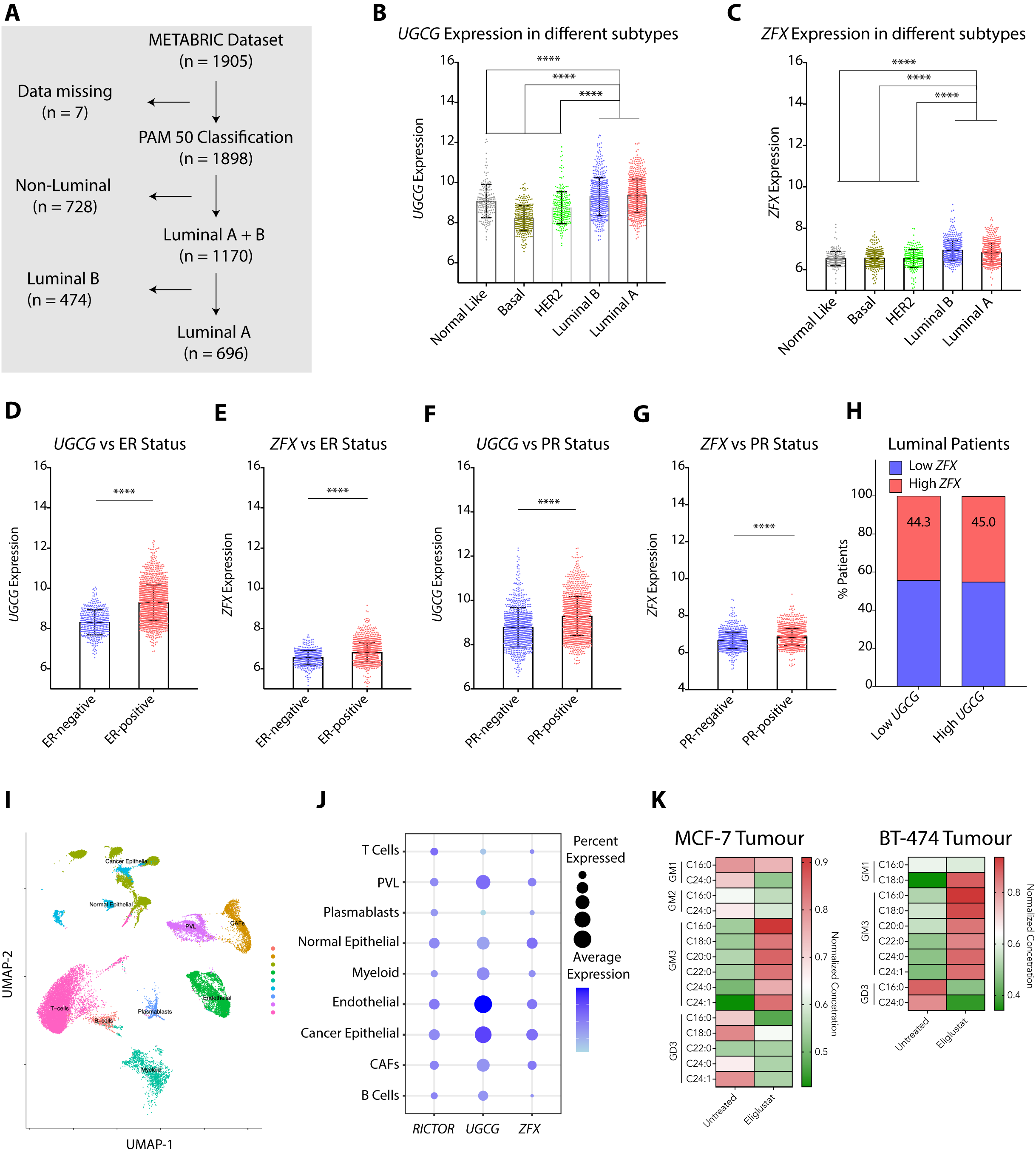

Supplement: S6 Fig — (A) Schematic showing the PAM50 classification of the METABRIC tumor dataset used for analysis. (B, C) Gene expression of UGCG (B) and ZFX (C) in different breast cancer subtypes (PAM50) of the METABRIC dataset confirms high expression of UGCG and ZFX in luminal subtypes compared to other subtypes. (D–G) Change in expression of UGCG (D, F) and ZFX (E, G) with respect to ER (D, E) and PR (F, G) status in breast tumors of the METABRIC dataset confirms high UGCG (D, F) and high ZFX (E, G) expression in ER+ and PR+ tumors. (H) Percentage of tumors having high expression of UGCG and ZFX among luminal subtype tumors in the METABRIC data set. (I) UMAP analysis of a single-cell RNA sequencing dataset of breast cancer patients displayed nine distinct clusters of cells. (J) Dot plots represent the average expression (color) and the proportion of cells expressing RICTOR, UGCG, and ZFX across all cell clusters. (K) Changes (Mean ± SEM, n = 3–5) in gangliosides in eliglustat-treated tumors compared to untreated MCF-7 and BT-474 tumors. Data among two groups were analyzed using an unpaired Student t test and among multiple groups using One-way ANOVA. p-value: *p < 0.05, **p < 0.01, ****p < 0.0001. Numerical data can be found in S6 Dataset. (S6_Fig.TIF) [file pbio.3003362.s006.tif]
