# Supplementary material for: The mTORC2 subunit RICTOR drives breast cancer progression by promoting ganglioside biosynthesis through transcriptional and epigenetic mechanisms
Source: PLoS Biol. 2025 Sep 11;23(9):e3003362. doi: 10.1371/journal.pbio.3003362 (PMC12425323; doi:10.1371/journal.pbio.3003362)
Supplement: S1 Table — (S1_Table.DOCX) [file pbio.3003362.s007.docx]

**S1 Table.** Table showing the clinical and pathological information of luminal breast cancer (ER^+^ PR^+^ HER2^-^) female patients of Indian origin included in this study.
