# Supplementary material for: The mTORC2 subunit RICTOR drives breast cancer progression by promoting ganglioside biosynthesis through transcriptional and epigenetic mechanisms
Source: PLoS Biol. 2025 Sep 11;23(9):e3003362. doi: 10.1371/journal.pbio.3003362 (PMC12425323; doi:10.1371/journal.pbio.3003362)
Supplement: S2 Table — (S2_Table.DOCX) [file pbio.3003362.s008.docx]

**S2 Table.** Table showing the immunohistochemistry data of tissue microarray from tumor samples of Indian, female breast cancer patients showing the score for UGCG and ZFX staining.
