# Supplementary material for: The mTORC2 subunit RICTOR drives breast cancer progression by promoting ganglioside biosynthesis through transcriptional and epigenetic mechanisms
Source: PLoS Biol. 2025 Sep 11;23(9):e3003362. doi: 10.1371/journal.pbio.3003362 (PMC12425323; doi:10.1371/journal.pbio.3003362)
Supplement: S3 Table — (S3_Table.DOCX) [file pbio.3003362.s009.docx]

| **S3 Table.** List of primers (human) used for validation of endogenous gene expression by Real-Time PCR. | | |
| --- | --- | --- |
| **Gene Name** | **Forward primer (5' to 3')** | **Reverse primer (5' to 3')** |
| *UGCG* | GAATGGCCGTCTTCGGGTTC | CACAAGAGAAGACACCTGGGAG |
| *GBA1* | GATACCAAGCTCAAGATACCC | GGTCTGGTGGTAGATGTCTC |
| *ELF1* | TGTTGTCCAACAGAACGACCT | GGAAAAATAGCTGGATCACCA |
| *ZFX* | GGATGATGCTGGCAAAATAGAAC | CAGTTCCACCTAAGTCATCTTC |
| *CTCF* | GACGAGTACCTGTGTGTGTG | CCAGTGTGAGCTTTGCAGTTA |
| *β-ACTIN* | ATTGGCAATGAGCGGTTCC | GGTAGAGTTTCGTGGATGCCACA |
