# Supplementary material for: The mTORC2 subunit RICTOR drives breast cancer progression by promoting ganglioside biosynthesis through transcriptional and epigenetic mechanisms
Source: PLoS Biol. 2025 Sep 11;23(9):e3003362. doi: 10.1371/journal.pbio.3003362 (PMC12425323; doi:10.1371/journal.pbio.3003362)
Supplement: S4 Table — (S4_Table.DOCX) [file pbio.3003362.s010.docx]

| **S4 Table.** List of primers (human) targeting UGCG promoter used for validation of ChiP events. | | |
| --- | --- | --- |
| **Antibody** | **Forward primer (5' to 3')** | **Reverse primer (5' to 3')** |
| *ZFX/ H3K4Me3* | CTCTCCGGTCCCTTTTGATC | CGGAGCTTGTCTAGAAATCCA |
| For both *ZFX* and *H3K4Me3* ChIP, same primer was used as it was the common region of UGCG promoter for enrichment of ZFX and H3K4Me3. | | |
