# Supplementary material for: The mTORC2 subunit RICTOR drives breast cancer progression by promoting ganglioside biosynthesis through transcriptional and epigenetic mechanisms
Source: PLoS Biol. 2025 Sep 11;23(9):e3003362. doi: 10.1371/journal.pbio.3003362 (PMC12425323; doi:10.1371/journal.pbio.3003362)
Supplement: S5 Table — (S5_Table.DOCX) [file pbio.3003362.s011.docx]

| **S5 Table.** Table showing parameters for estimation of gangliosides and globosides from cell lines. | | | |
| --- | --- | --- | --- |
| **Ganglioside Species** | **Precursor ion (Q1)** | **Product ion (Q3)** | **Collision Energy (V)** |
| GM3-d3 | 1182.7 | 290.1 | -60 |
| GD3-d3 | 736.3 | 290.1 | -45 |
| GM1-d3 | 1547.8 | 290.1 | -80 |
| d18:1/C16:0 GM1 | 1516.8 | 290.1 | -80 |
| d18:1/C18:0 GM1 | 1544.8 | 290.1 | -80 |
| d18:1/C20:0 GM1 | 1572.9 | 290.1 | -80 |
| d18:1/C22:0 GM1 | 1600.9 | 290.1 | -80 |
| d18:1/C24:0 GM1 | 1628.9 | 290.1 | -80 |
| d18:1/C24:1 GM1 | 1626.9 | 290.1 | -80 |
| d18:1/C16:0 GM2 | 1354.7 | 290.1 | -75 |
| d18:1/C18:0 GM2 | 1382.8 | 290.1 | -75 |
| d18:1/C20:0 GM2 | 1410.8 | 290.1 | -75 |
| d18:1/C22:0 GM2 | 1438.8 | 290.1 | -75 |
| d18:1/C24:0 GM2 | 1466.9 | 290.1 | -75 |
| d18:1/C24:1 GM2 | 1464.9 | 290.1 | -75 |
| d18:1/C16:0 GM3 | 1151.7 | 290.1 | -60 |
| d18:1/C18:0 GM3 | 1179.7 | 290.1 | -60 |
| d18:1/C20:0 GM3 | 1207.7 | 290.1 | -60 |
| d18:1/C22:0 GM3 | 1235.8 | 290.1 | -60 |
| d18:1/C24:0 GM3 | 1263.8 | 290.1 | -60 |
| d18:1/C24:1 GM3 | 1261.8 | 290.1 | -60 |
| d18:1/C16:0 GD2 | 822.5 | 290.1 | -50 |
| d18:1/C18:0 GD2 | 836.5 | 290.1 | -50 |
| d18:1/C20:0 GD2 | 850.5 | 290.1 | -50 |
| d18:1/C22:0 GD2 | 864.5 | 290.1 | -50 |
| d18:1/C24:0 GD2 | 878.5 | 290.1 | -50 |
| d18:1/C24:1 GD2 | 876.5 | 290.1 | -50 |
| d18:1/C16:0 GD3 | 720.9 | 290.1 | -45 |
| d18:1/C18:0 GD3 | 734.9 | 290.1 | -45 |
| d18:1/C20:0 GD3 | 748.9 | 290.1 | -45 |
| d18:1/C22:0 GD3 | 762.9 | 290.1 | -45 |
| d18:1/C24:0 GD3 | 776.2 | 290.1 | -45 |
| d18:1/C24:1 GD3 | 774.2 | 290.1 | -45 |
