# Supplementary material for: The mTORC2 subunit RICTOR drives breast cancer progression by promoting ganglioside biosynthesis through transcriptional and epigenetic mechanisms
Source: PLoS Biol. 2025 Sep 11;23(9):e3003362. doi: 10.1371/journal.pbio.3003362 (PMC12425323; doi:10.1371/journal.pbio.3003362)
Supplement: S1 Raw images — (PDF) [file pbio.3003362.s024.pdf]

Fig 1

I

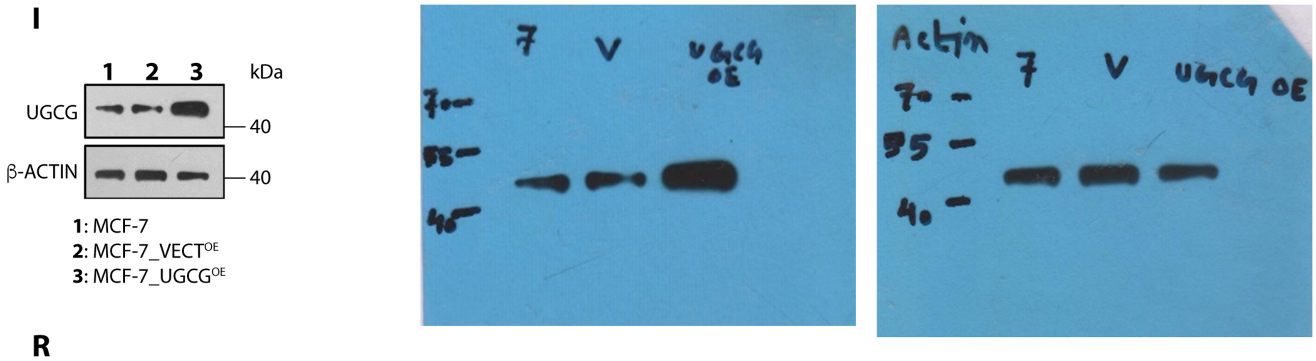

1 2 3 kDa
UGCG
β-ACTIN
40
40
1: MCF-7
2: MCF-7_VECT^OE
3: MCF-7_UGCG^OE

R

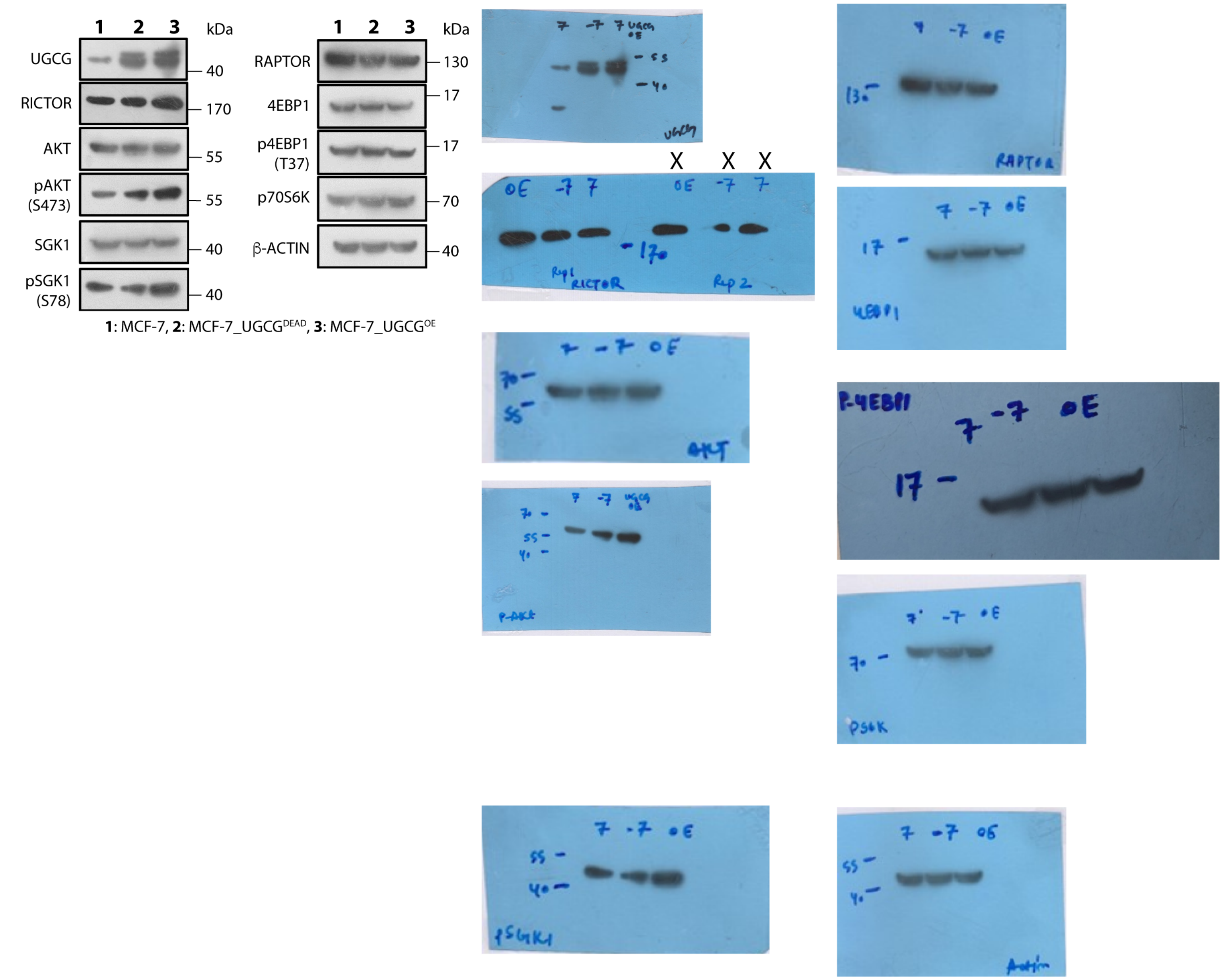

1 2 3 kDa
UGCG 40
RICTOR 170
AKT 55
pAKT (S473) 55
SGK1 40
pSGK1 (S78) 40
RAPTOR 130
4EBP1 17
p4EBP1 (T37) 17
p70S6K 70
β-ACTIN 40
1: MCF-7, 2: MCF-7_UGCG^DEAD, 3: MCF-7_UGCG^OE
X X X

S

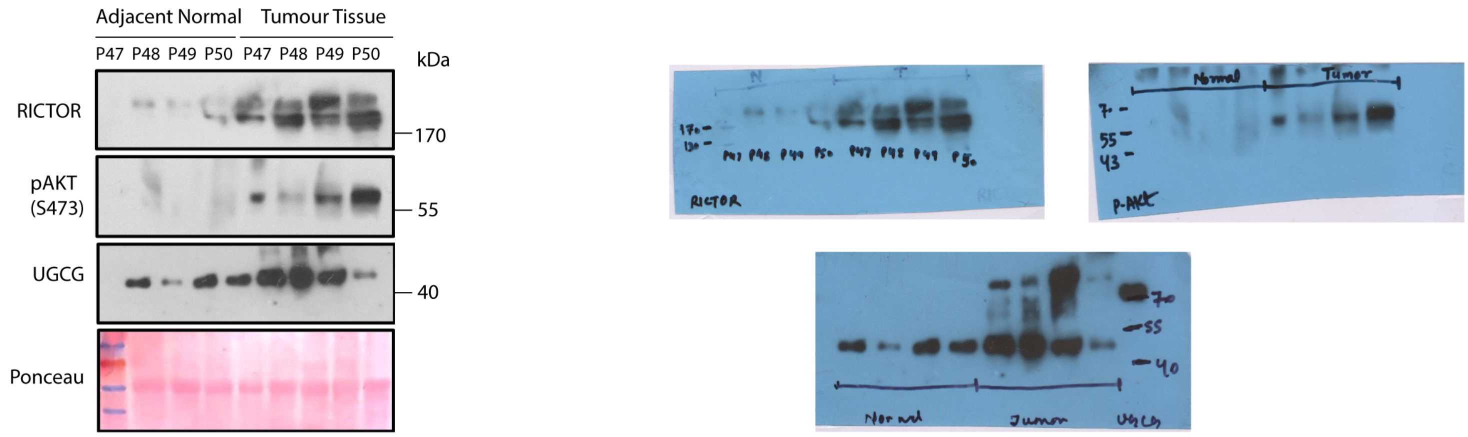

Adjacent Normal
Tumour Tissue
P47 P48 P49 P50 P47 P48 P49 P50
kDa
RICTOR 170
pAKT (S473) 55
UGCG 40
Ponceau

Fig 2

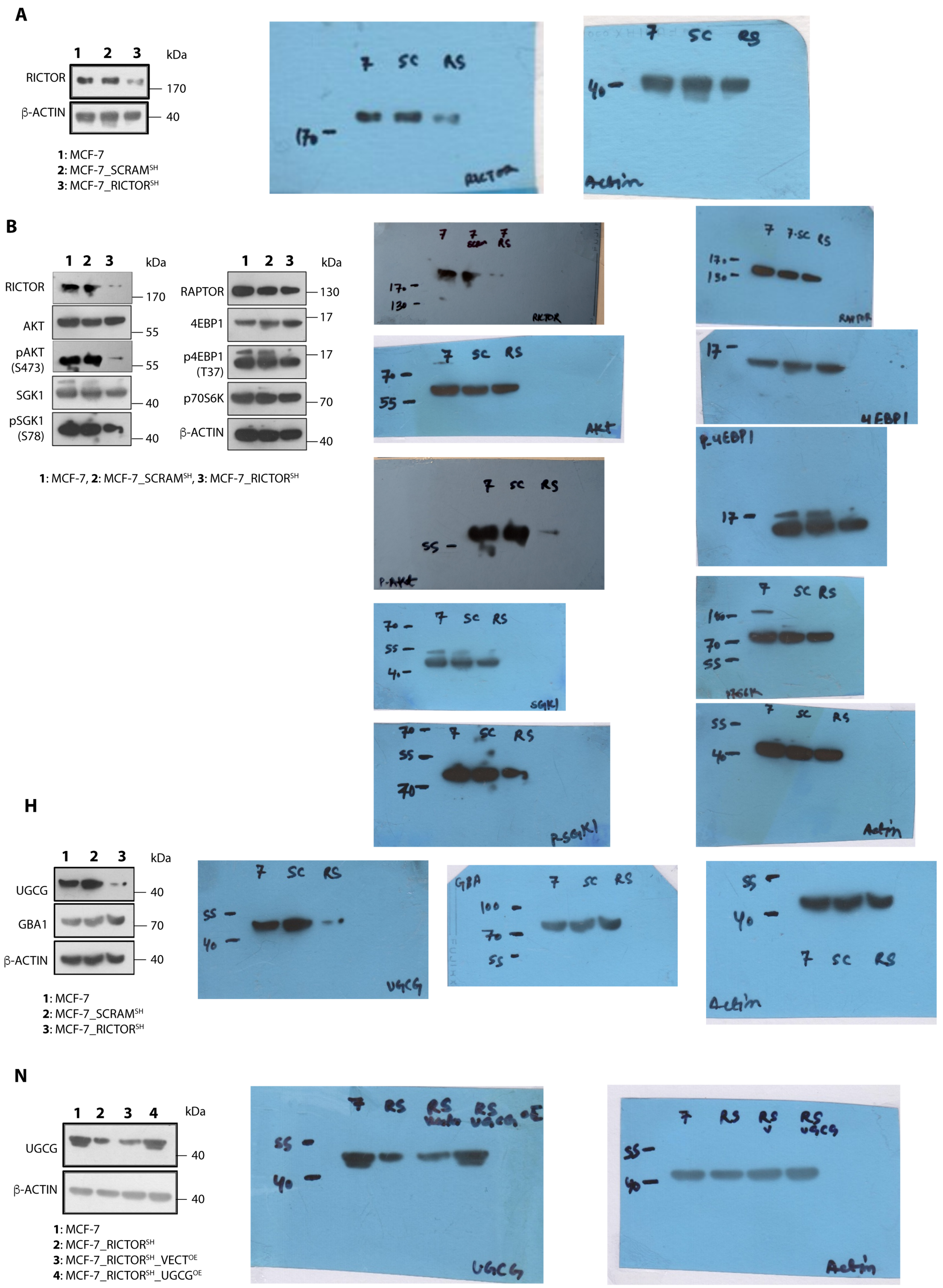
A
1 2 3 kDa
RICTOR 170
β-ACTIN 40
1: MCF-7
2: MCF-7_SCRAMSH
3: MCF-7_RICTORSH
B
1 2 3 kDa
RICTOR 170
AKT 55
pAKT (S473) 55
SGK1 40
pSGK1 (S78) 40
RAPTOR 130
4EBP1 17
p4EBP1 (T37) 17
p70S6K 70
β-ACTIN 40
1: MCF-7, 2: MCF-7_SCRAMSH, 3: MCF-7_RICTORSH
H
1 2 3 kDa
UGCG 40
GBA1 70
β-ACTIN 40
1: MCF-7
2: MCF-7_SCRAMSH
3: MCF-7_RICTORSH
N
1 2 3 4 kDa
UGCG 40
β-ACTIN 40
1: MCF-7
2: MCF-7_RICTORSH
3: MCF-7_RICTORSH_VECTOE
4: MCF-7_RICTORSH_UGCGOE

Fig 3

D

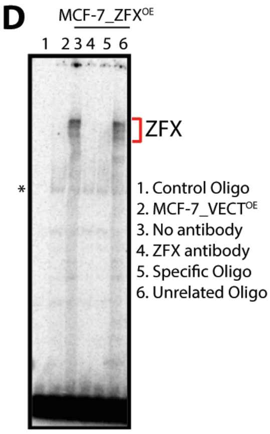

1. Control Oligo
2. MCF-7_VECT^{OE}
3. No antibody
4. ZFX antibody
5. Specific Oligo
6. Unrelated Oligo

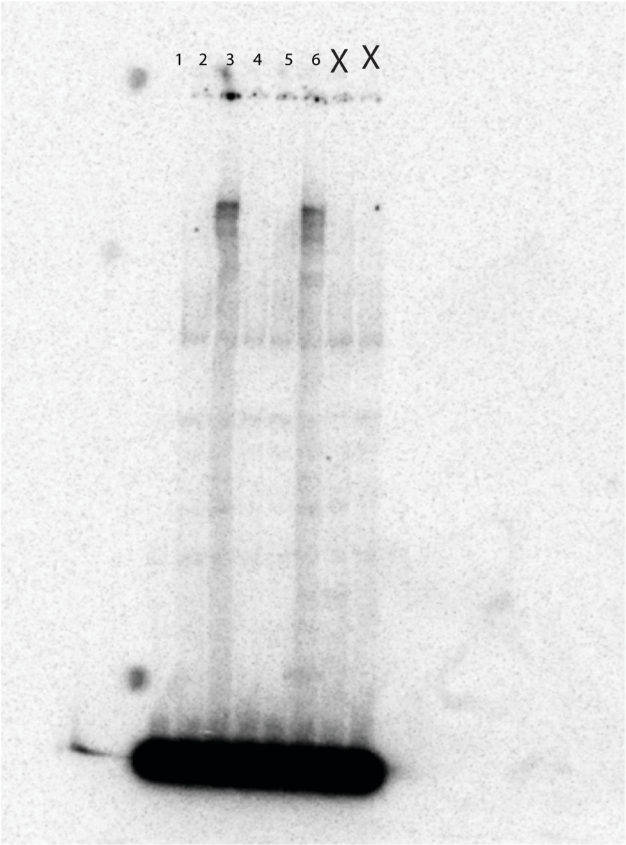

E

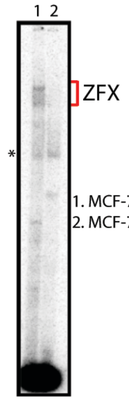

1. MCF-7_SCRAM^{SH}
2. MCF-7_RICTOR^{SH}

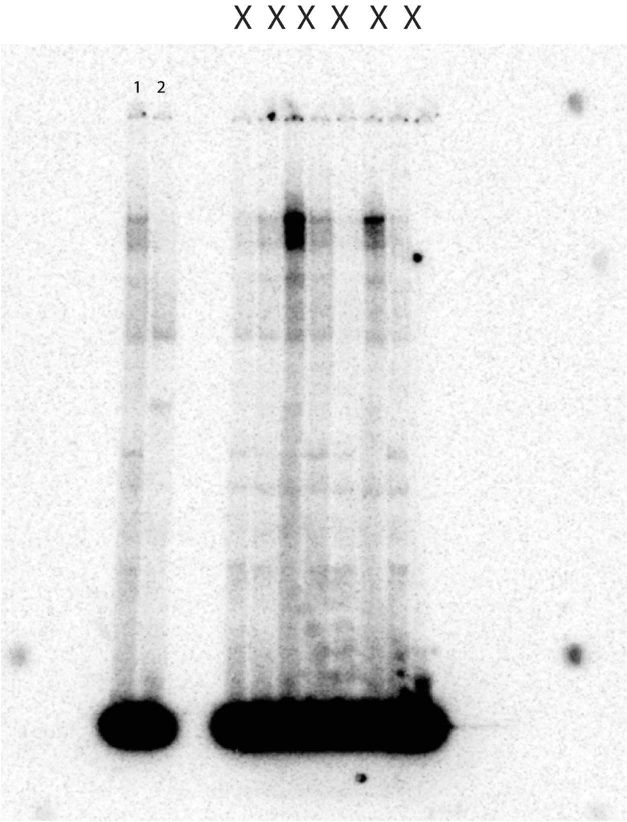

Fig 3

F

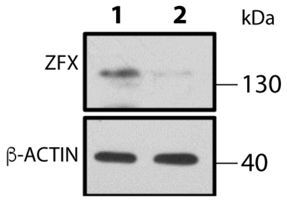

1: MCF-7
2: MCF-7_RICTOR^{SH}

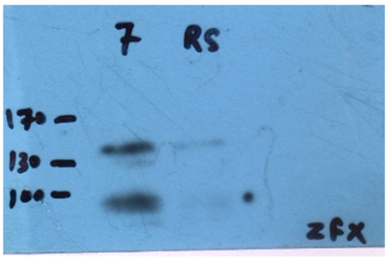

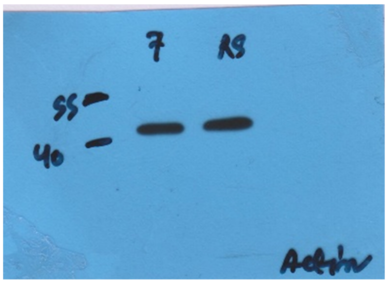

H

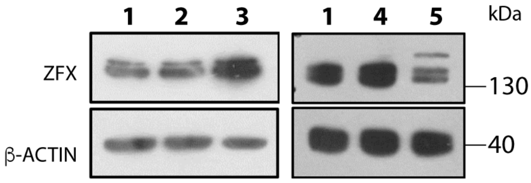

1: MCF-7, **2**: MCF-7_VECT^{OE}, **3**: MCF-7_ZFX^{OE},
4: MCF-7_SCRAM^{SL}, **5**: MCF-7_ZFX^{SL}

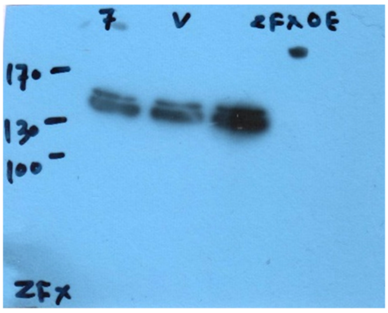

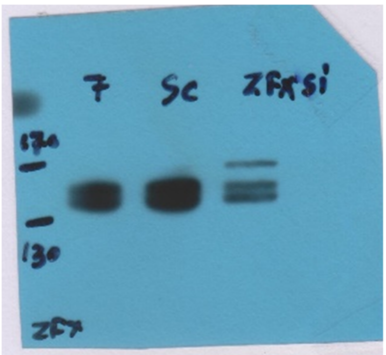

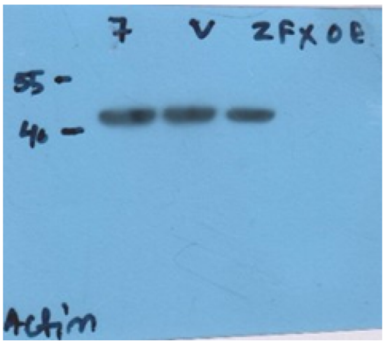

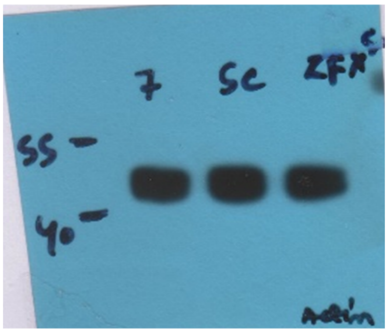

J

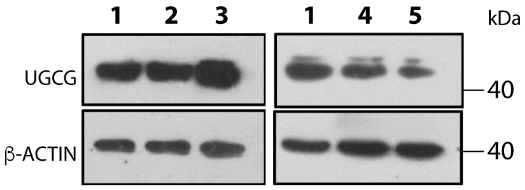

1: MCF-7, **2**: MCF-7_VECT^{OE}, **3**: MCF-7_ZFX^{OE},
4: MCF-7_SCRAM^{SL}, **5**: MCF-7_ZFX^{SL}

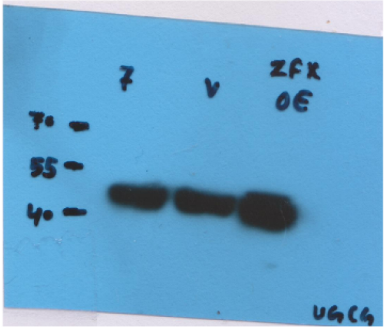

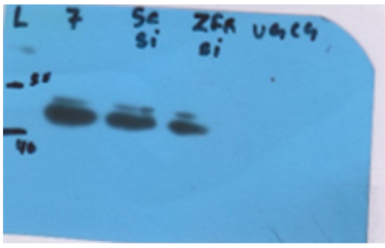

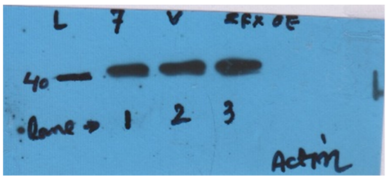

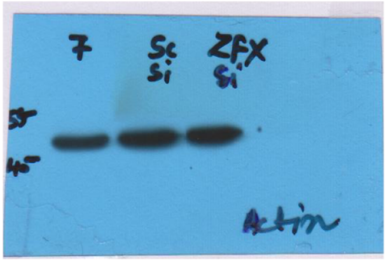

Fig 4

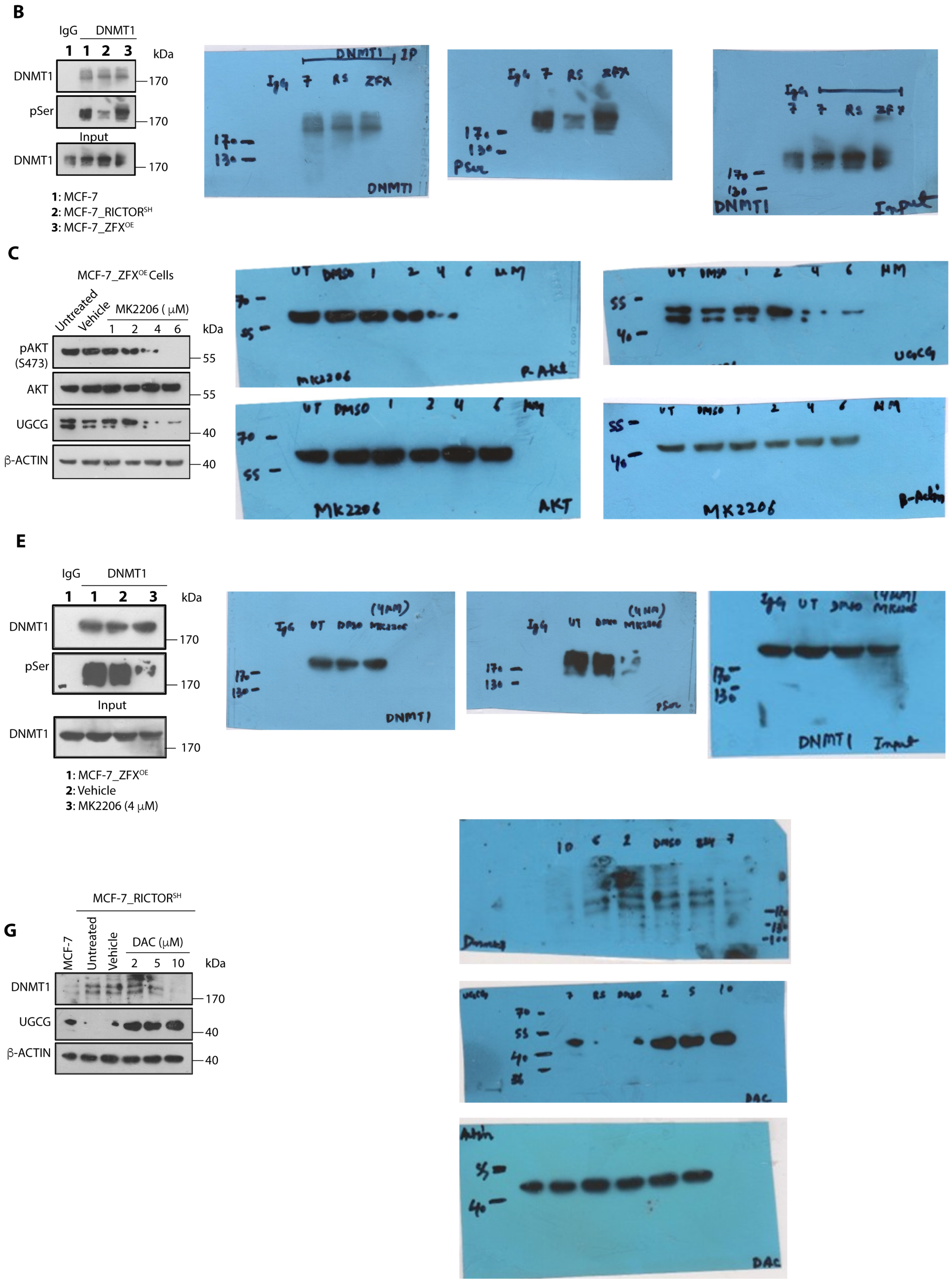
B
IgG
DNMT1
1 1 2 3
kDa
DNMT1
pSer
Input
DNMT1
170
1: MCF-7
2: MCF-7_RICTOR^SH
3: MCF-7_ZFX^OE
C
MCF-7_ZFX^OE Cells
Untreated
Vehicle
MK2206 (µM)
1 2 4 6
kDa
pAKT (S473)
AKT
UGCG
β-ACTIN
55
40
E
IgG
DNMT1
1 1 2 3
kDa
DNMT1
pSer
Input
DNMT1
170
1: MCF-7_ZFX^OE
2: Vehicle
3: MK2206 (4 µM)
G
MCF-7_RICTOR^SH
MCF-7
Untreated
Vehicle
DAC (µM)
2 5 10
kDa
DNMT1
UGCG
β-ACTIN
170
40

Fig 4

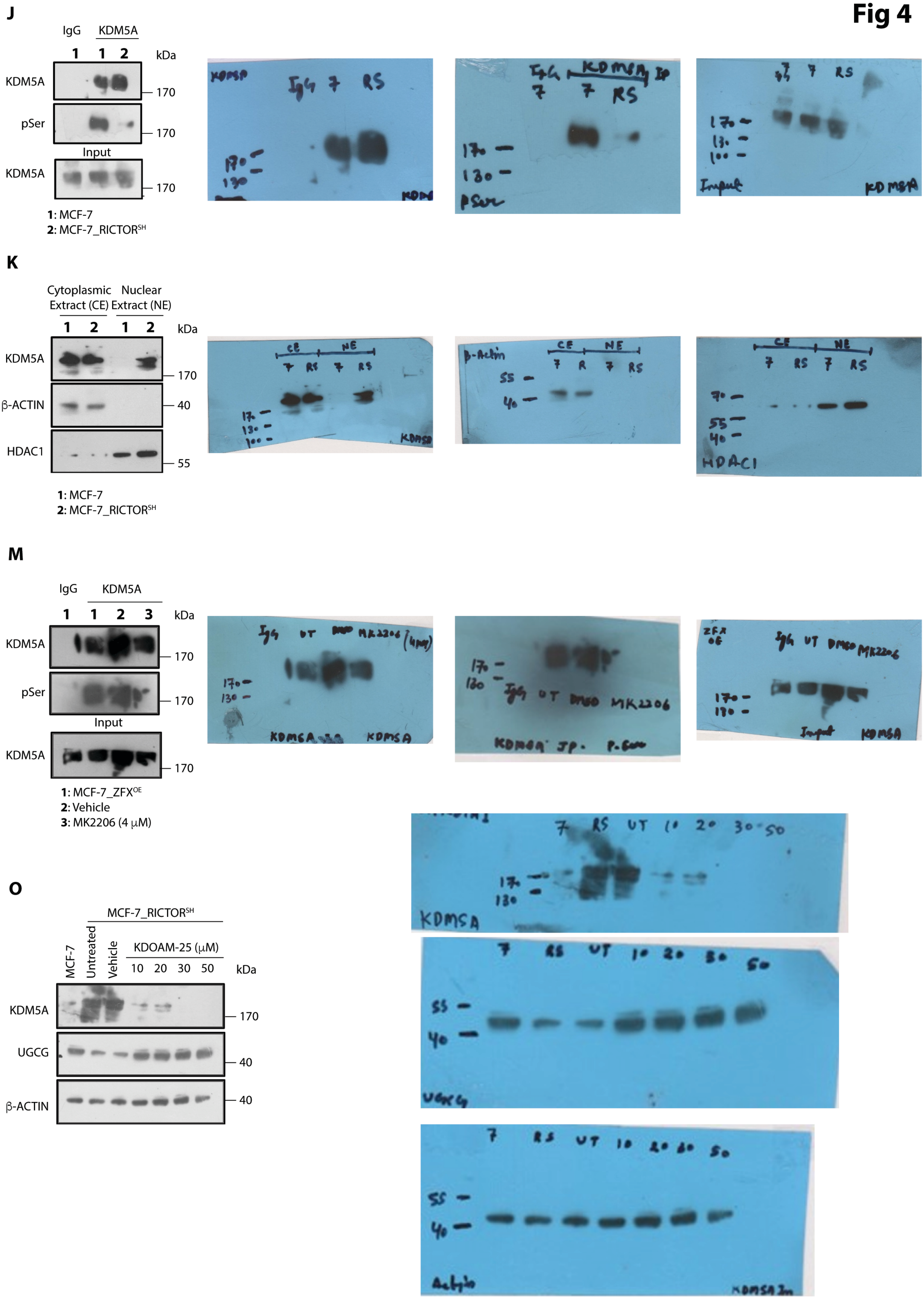
J
IgG
KDM5A
1
1
2
kDa
KDM5A
pSer
Input
170
1: MCF-7
2: MCF-7_RICTOR^SH
K
Cytoplasmic Extract (CE)
Nuclear Extract (NE)
1
2
kDa
KDM5A
β-ACTIN
HDAC1
170
40
55
1: MCF-7
2: MCF-7_RICTOR^SH
M
IgG
KDM5A
1
2
3
kDa
KDM5A
pSer
Input
170
1: MCF-7_ZFX^OE
2: Vehicle
3: MK2206 (4 μM)
O
MCF-7_RICTOR^SH
MCF-7
Untreated
Vehicle
KDOAM-25 (μM)
10
20
30
50
kDa
KDM5A
UGCG
β-ACTIN
170
40

Fig 5

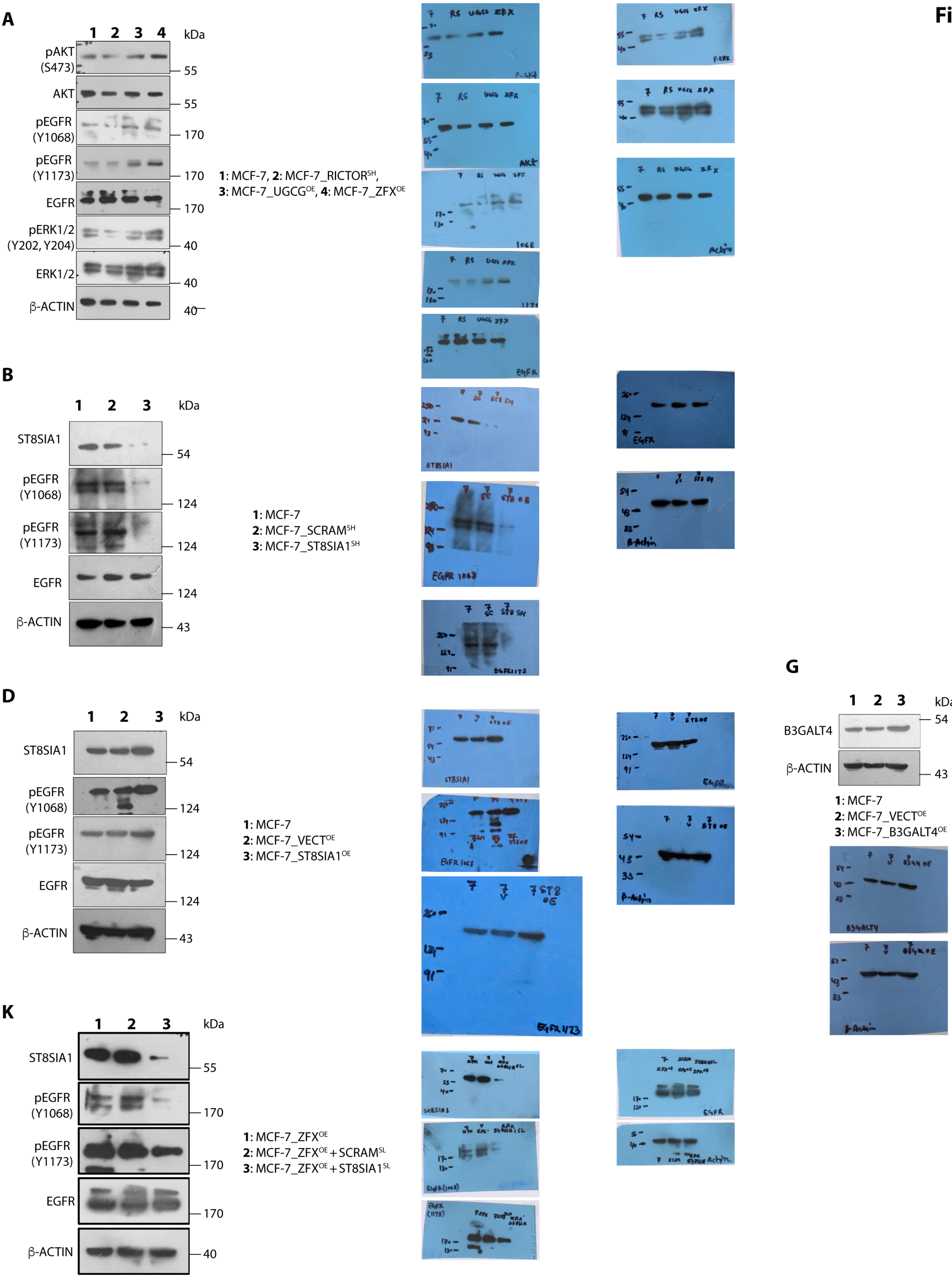

A
1 2 3 4 kDa
pAKT (S473)
AKT
pEGFR (Y1068)
pEGFR (Y1173)
EGFR
pERK1/2 (Y202, Y204)
ERK1/2
β-ACTIN
55
55
170
170
170
40
40
40
1: MCF-7, 2: MCF-7_RICTOR^SH,
3: MCF-7_UGCG^OE, 4: MCF-7_ZFX^OE
B
1 2 3 kDa
ST8SIA1
pEGFR (Y1068)
pEGFR (Y1173)
EGFR
β-ACTIN
54
124
124
124
43
1: MCF-7
2: MCF-7_SCRAM^SH
3: MCF-7_ST8SIA1^SH
D
1 2 3 kDa
ST8SIA1
pEGFR (Y1068)
pEGFR (Y1173)
EGFR
β-ACTIN
54
124
124
124
43
1: MCF-7
2: MCF-7_VECT^OE
3: MCF-7_ST8SIA1^OE
G
1 2 3 kDa
B3GALT4
β-ACTIN
54
43
1: MCF-7
2: MCF-7_VECT^OE
3: MCF-7_B3GALT4^OE
K
1 2 3 kDa
ST8SIA1
pEGFR (Y1068)
pEGFR (Y1173)
EGFR
β-ACTIN
55
170
170
170
40
1: MCF-7_ZFX^OE
2: MCF-7_ZFX^OE + SCRAM^SL
3: MCF-7_ZFX^OE + ST8SIA1^SL

S1 Fig

C

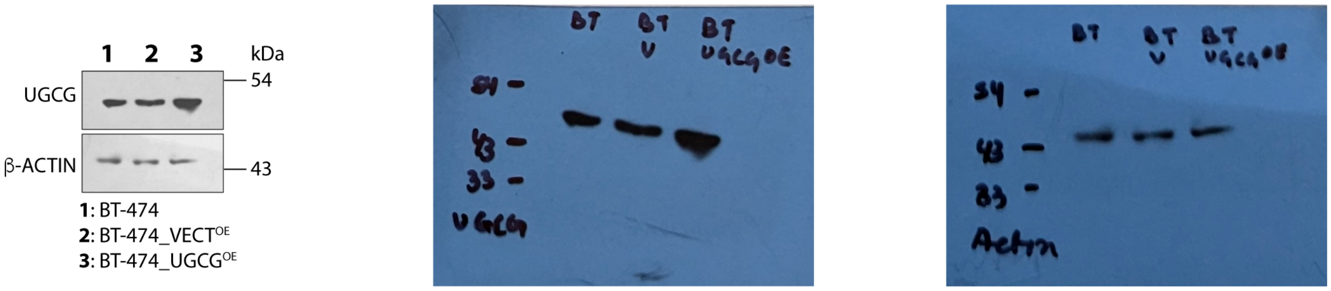

1 2 3 kDa
UGCG
54
β-ACTIN
43
1: BT-474
2: BT-474_VECT^OE
3: BT-474_UGCG^OE

J

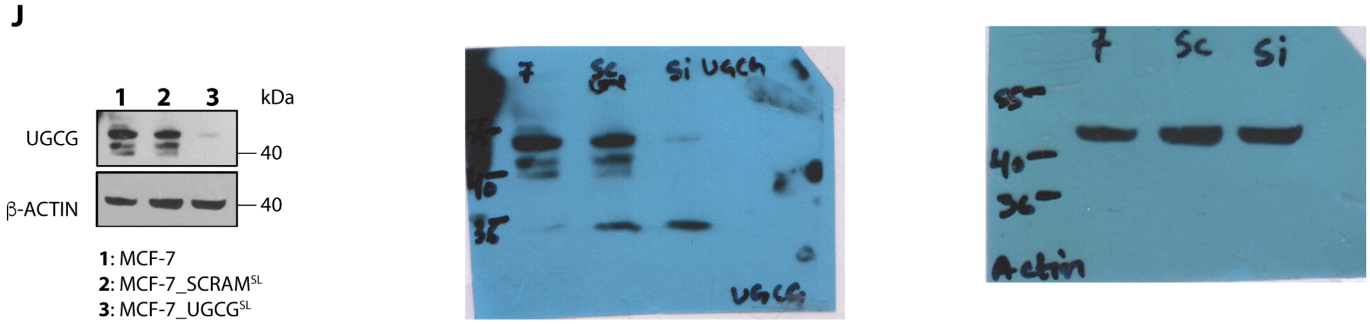

1 2 3 kDa
UGCG
40
β-ACTIN
40
1: MCF-7
2: MCF-7_SCRAM^SL
3: MCF-7_UGCG^SL

L

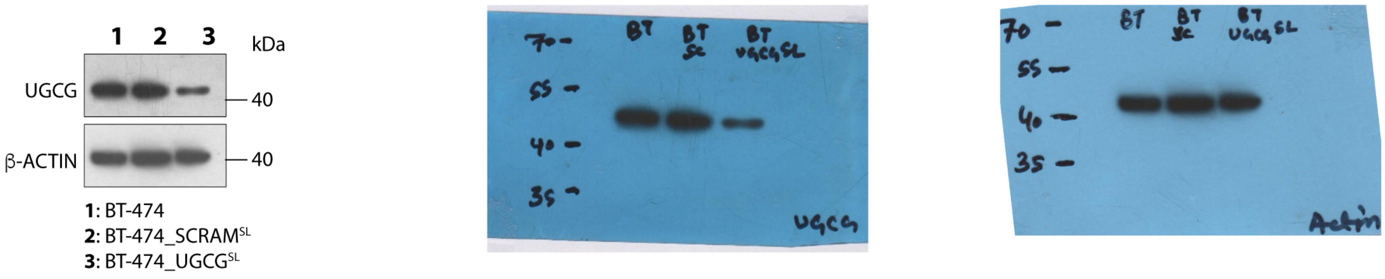

1 2 3 kDa
UGCG
40
β-ACTIN
40
1: BT-474
2: BT-474_SCRAM^SL
3: BT-474_UGCG^SL

Q

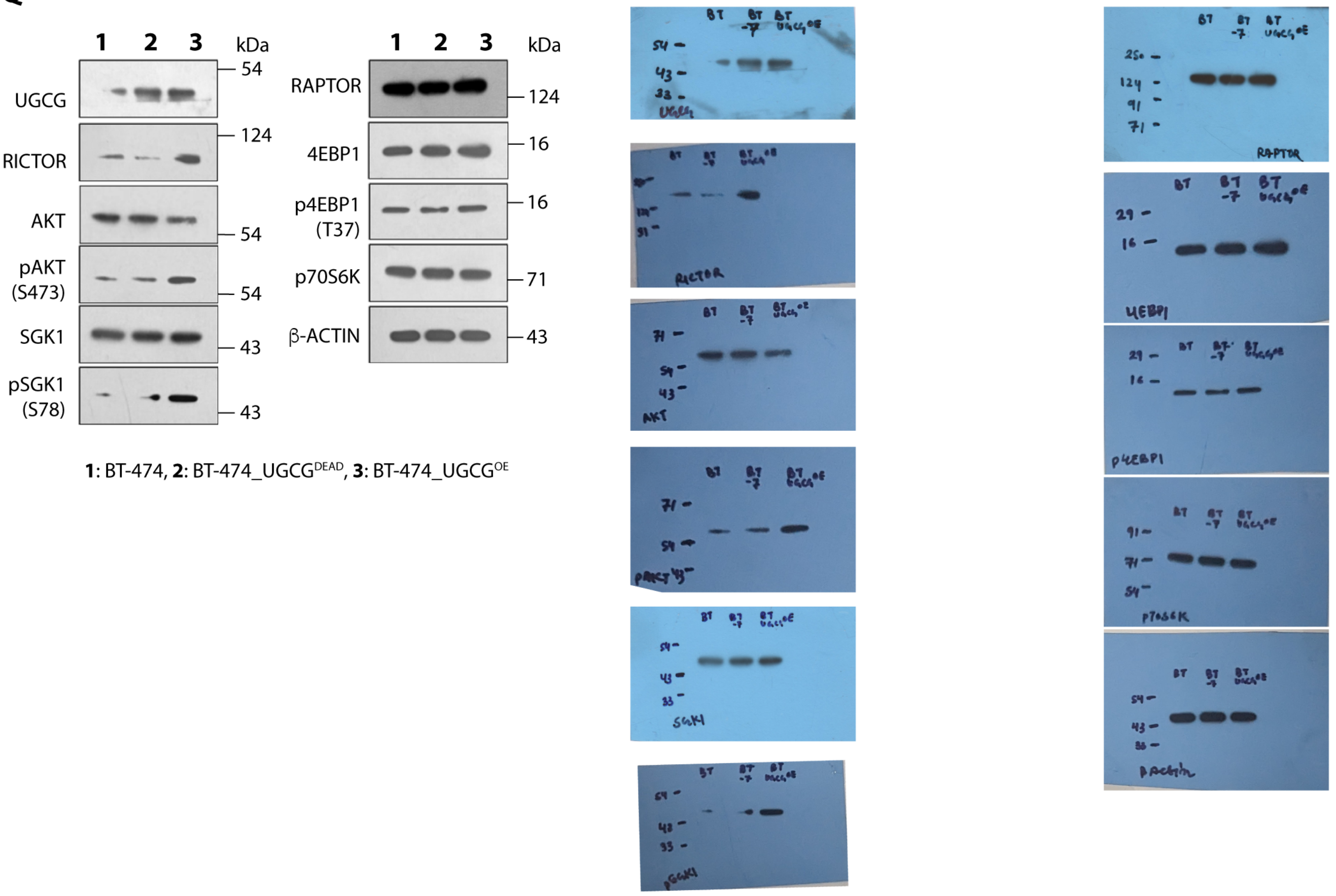

1 2 3 kDa
UGCG
54
RICTOR
124
AKT
54
pAKT (S473)
54
SGK1
43
pSGK1 (S78)
43
RAPTOR
124
4EBP1
16
p4EBP1 (T37)
16
p70S6K
71
β-ACTIN
43
1: BT-474, 2: BT-474_UGCG^DEAD, 3: BT-474_UGCG^OE

S2 Fig

A

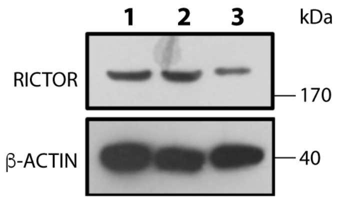

1: BT-474
2: BT-474_SCRAM^{SH}
3: BT-474_RICTOR^{SH}

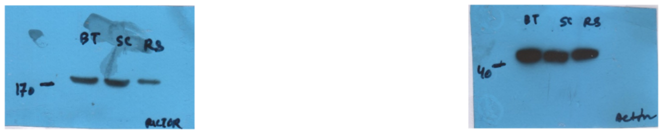

B

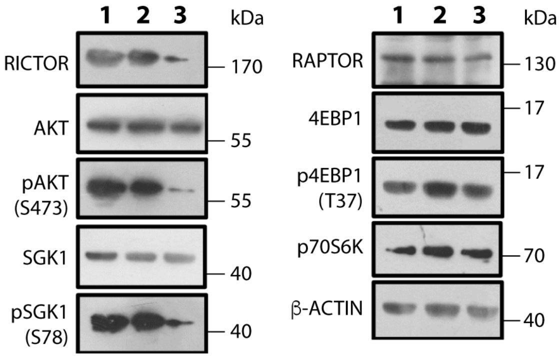

1: BT-474, **2**: BT-474_SCRAM^{SH}, **3**: BT-474_RICTOR^{SH}

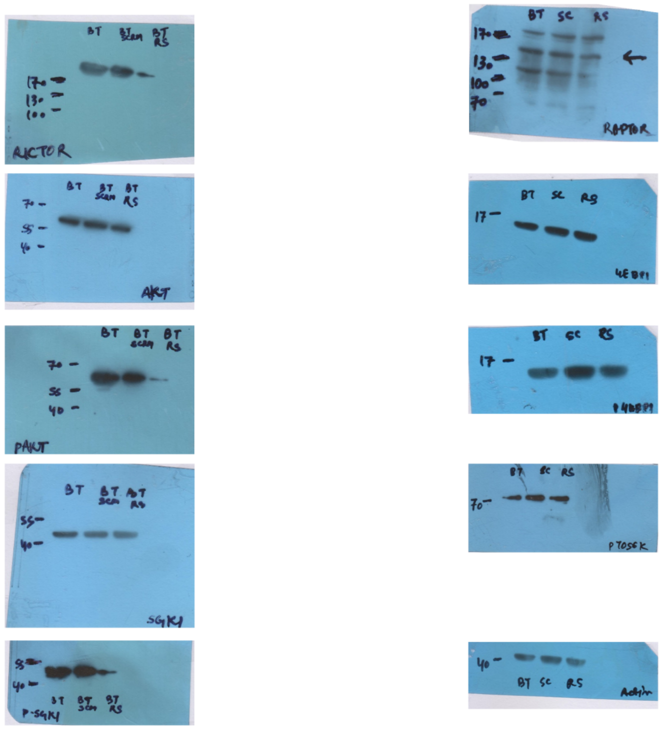

G

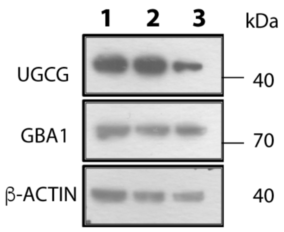

1: BT-474
2: BT-474_SCRAM^{SH}
3: BT-474_RICTOR^{SH}

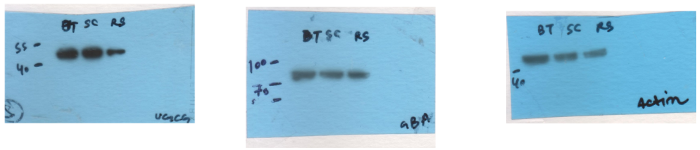

K

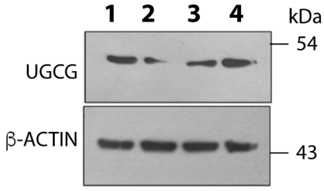

1: BT-474
2: BT-474_RICTOR^{SH}
3: BT-474_RICTOR^{SH}_VECT^{OE}
4: BT-474_RICTOR^{SH}_UGCG^{OE}

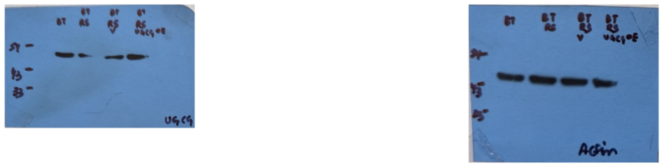

N

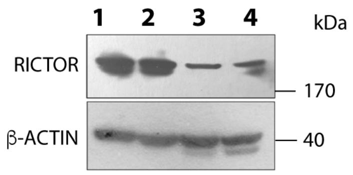

1: HCT-116
2: HCT-116_SCRAM^{SH}
3: HCT-116_RICTOR$^{SH1\&2}$
4: HCT-116_RICTOR$^{SH3\&4}$

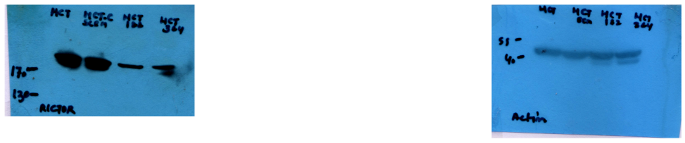

P

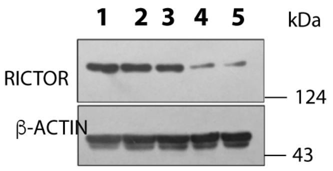

1: HEK-293
2: HEK-293_SCRAM^{SH}
3: HEK-293_RICTOR^{SH3}
4: HEK-293_RICTOR^{SH4}
5: HEK-293_RICTOR$^{SH3\&4}$

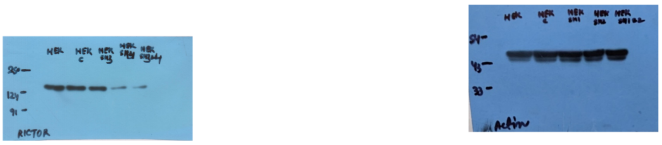

S3 Fig

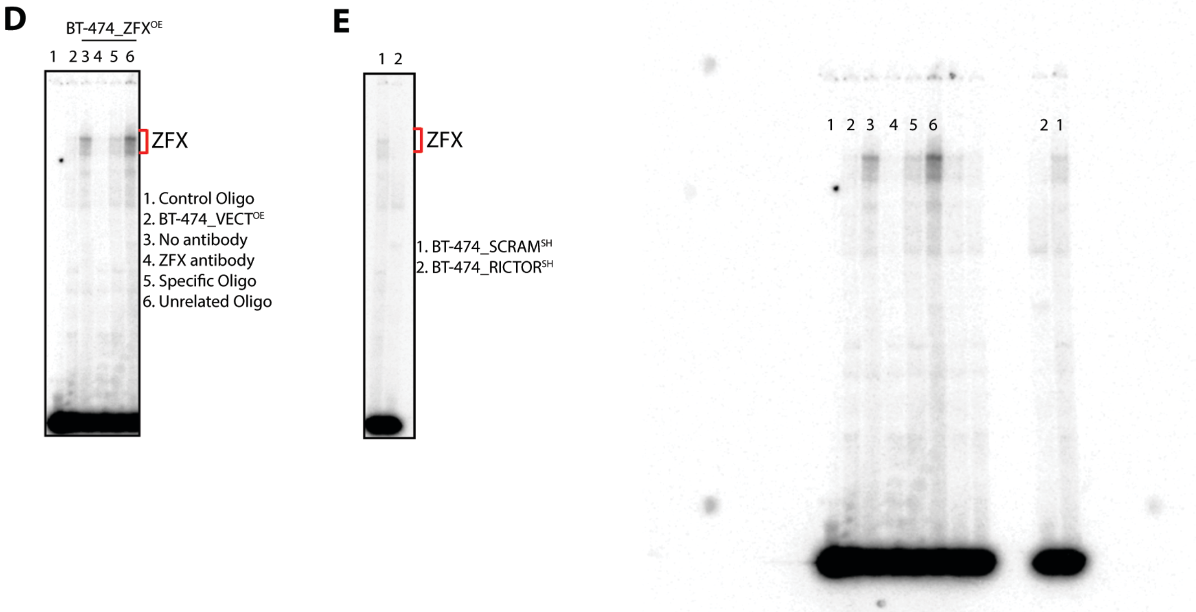
D
BT-474_ZFX^{OE}
1 2 3 4 5 6
ZFX
1. Control Oligo
2. BT-474_VECT^{OE}
3. No antibody
4. ZFX antibody
5. Specific Oligo
6. Unrelated Oligo
E
1 2
ZFX
1. BT-474_SCRAM^{SH}
2. BT-474_RICTOR^{SH}
1 2 3 4 5 6
2 1

S3 Fig

F

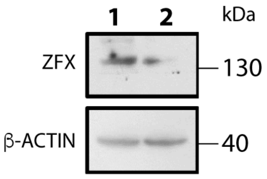

1: BT-474
2: BT-474_RICTOR^{SH}

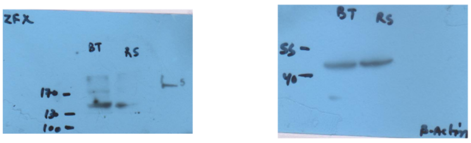

H

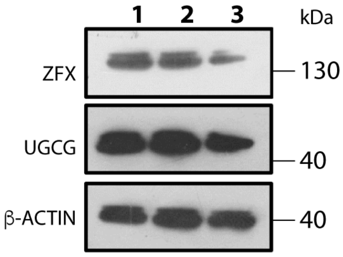

1: BT-474
2: BT-474_SCRAM^{SL}
3: BT-474_ZFX^{SL}

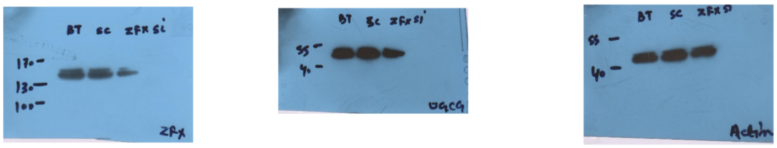

I

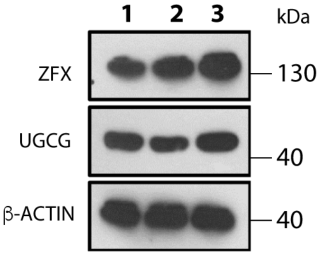

1: BT-474
2: BT-474_VECT^{OE}
3: BT-474_ZFX^{OE}

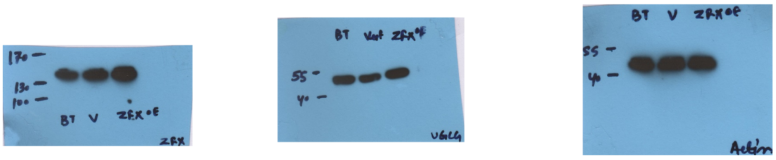

U

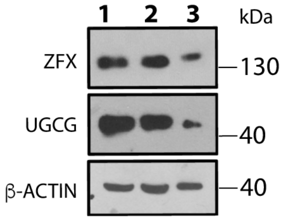

1: HCT-116, **2**: HCT-116_SCRAM^{SL}
3: HCT-116_ZFX^{SL}

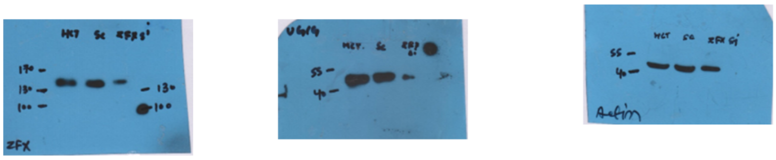

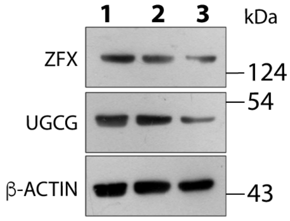

1: HEK-293, **2**: HEK-293_SCRAM^{SL}
3: HEK-293_ZFX^{SL}

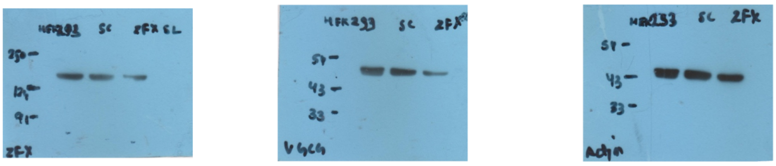

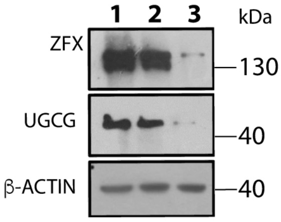

1: HepG2, **2**: HepG2_SCRAM^{SL}
3: HepG2_ZFX^{SL}

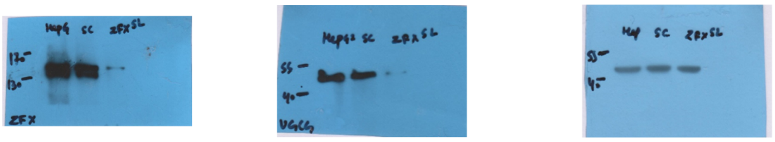

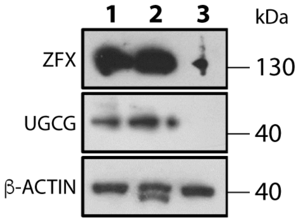

1: MDA-MB-453, **2**: MDA-MB-453_SCRAM^{SL}
3: MDA-MB-453_ZFX^{SL}

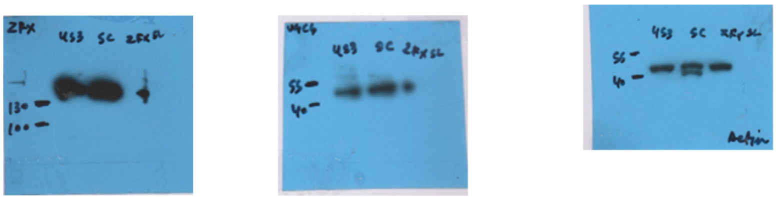

S4 Fig

A

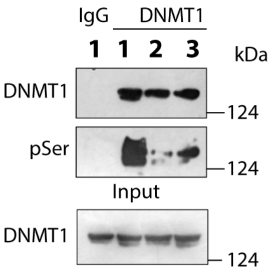

1: BT-474
2: BT-474_RICTOR^SH
3: BT-474_ZFX^OE

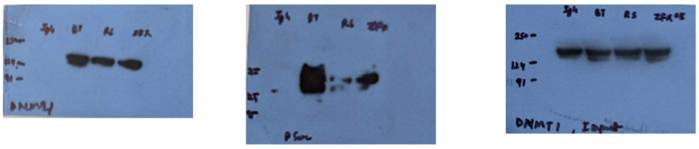

B

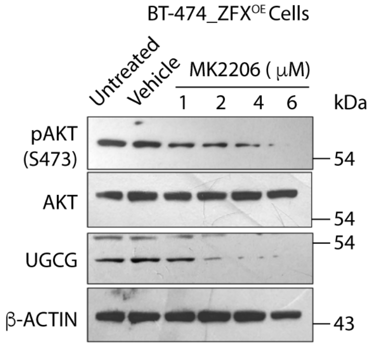

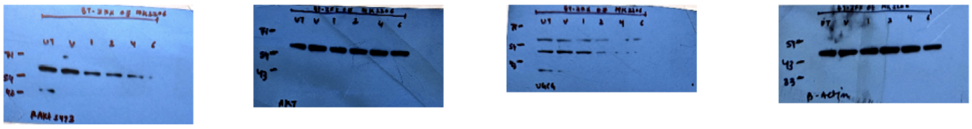

D

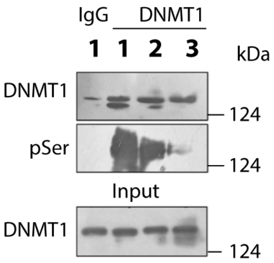

1: BT-474_ZFX^OE
2: Vehicle
3: MK2206 (4 μM)

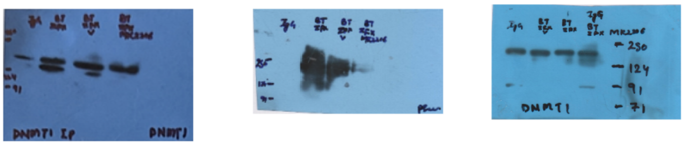

F

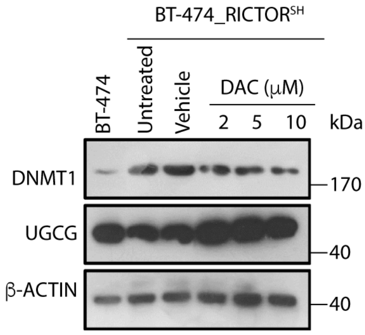

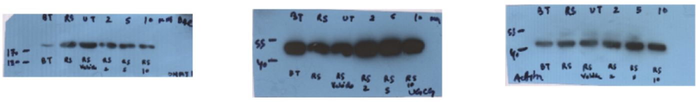

S4 Fig

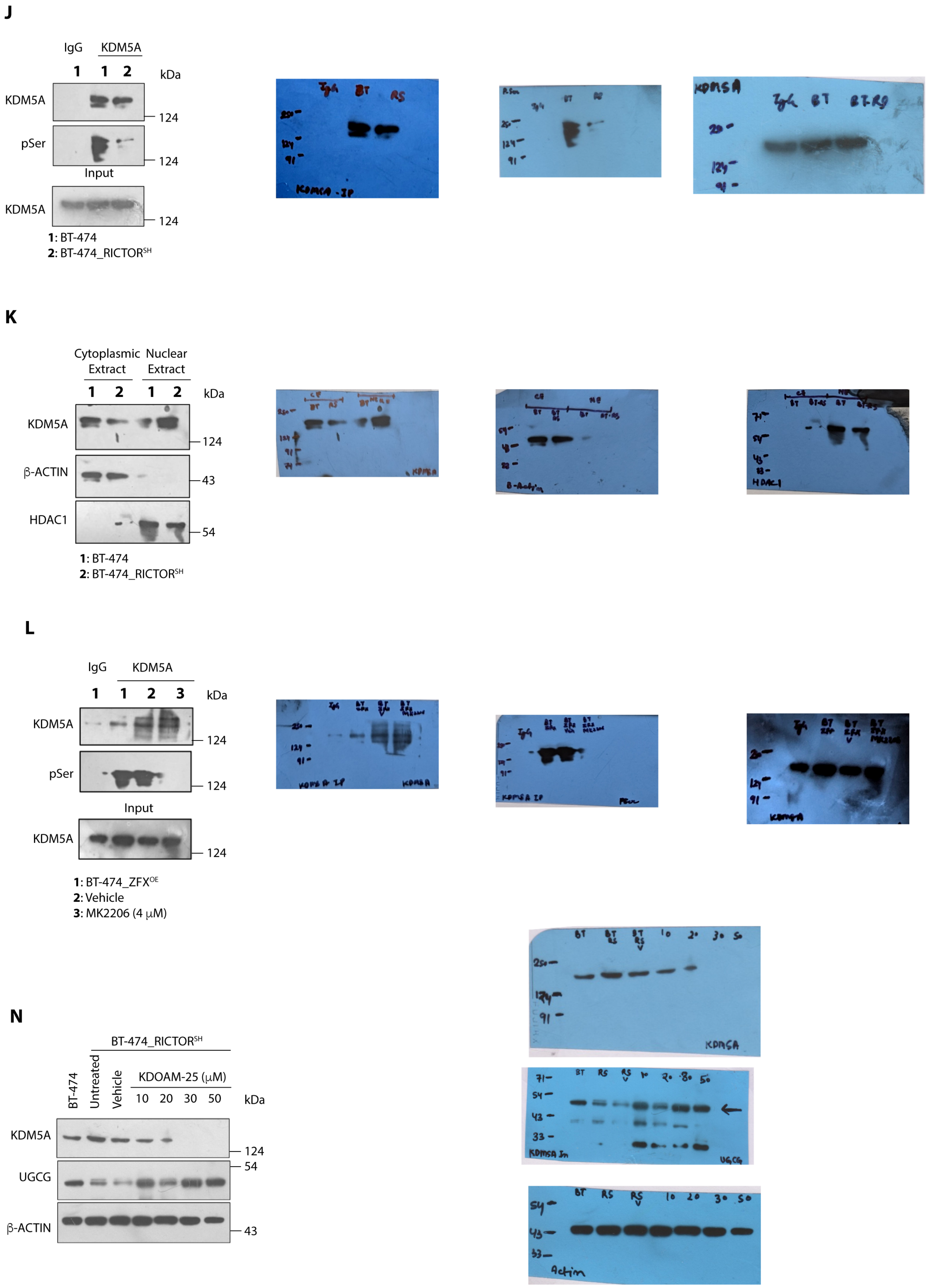
J
IgG
KDM5A
1 1 2
kDa
KDM5A
124
pSer
124
Input
KDM5A
124
1: BT-474
2: BT-474_RICTOR^SH
K
Cytoplasmic Extract
Nuclear Extract
1 2 1 2
kDa
KDM5A
124
β-ACTIN
43
HDAC1
54
1: BT-474
2: BT-474_RICTOR^SH
L
IgG
KDM5A
1 1 2 3
kDa
KDM5A
124
pSer
124
Input
KDM5A
124
1: BT-474_ZFX^OE
2: Vehicle
3: MK2206 (4 μM)
N
BT-474_RICTOR^SH
BT-474
Untreated
Vehicle
KDOAM-25 (μM)
10 20 30 50
kDa
KDM5A
124
UGCG
54
β-ACTIN
43

S5 Fig

A

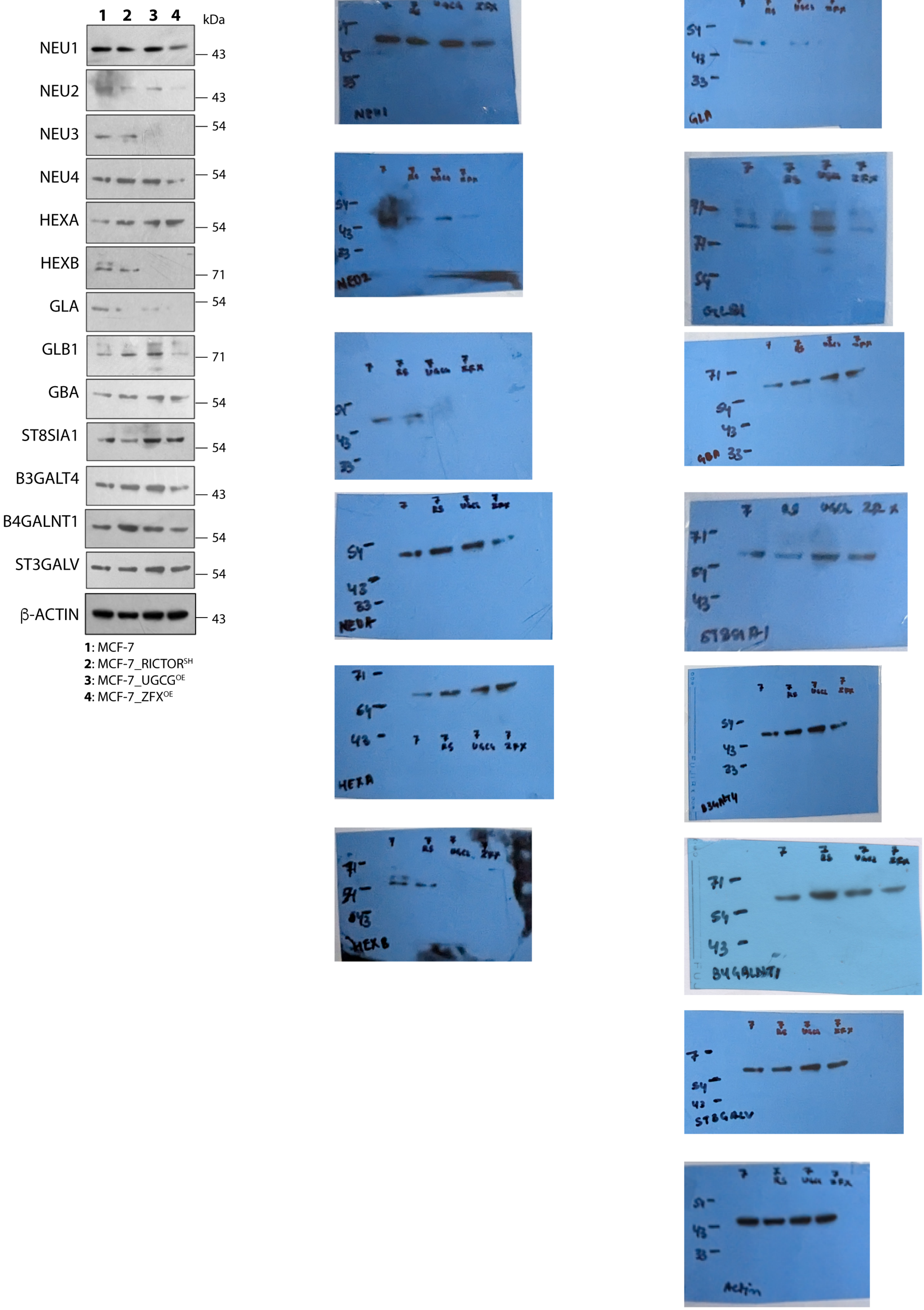
1 2 3 4
kDa
NEU1
43
NEU2
43
NEU3
54
NEU4
54
HEXA
54
HEXB
71
GLA
54
GLB1
71
GBA
54
ST8SIA1
54
B3GALT4
43
B4GALNT1
54
ST3GALV
54
β-ACTIN
43
1: MCF-7
2: MCF-7_RICTOR^SH
3: MCF-7_UGCG^OE
4: MCF-7_ZFX^OE

S5 Fig

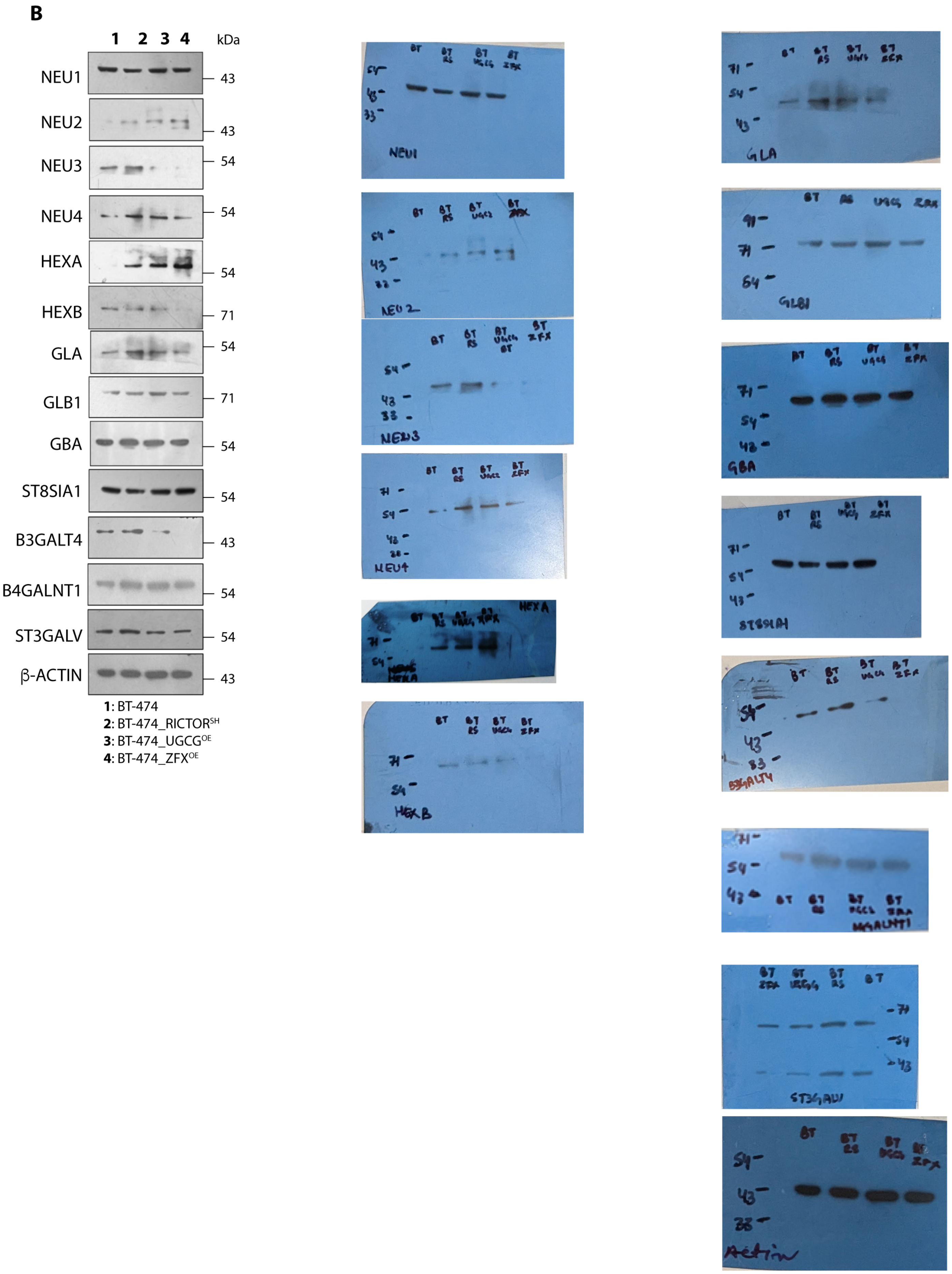

B
1 2 3 4
kDa
NEU1
43
NEU2
43
NEU3
54
NEU4
54
HEXA
54
HEXB
71
GLA
54
GLB1
71
GBA
54
ST8SIA1
54
B3GALT4
43
B4GALNT1
54
ST3GALV
54
β-ACTIN
43
1: BT-474
2: BT-474_RICTOR^SH
3: BT-474_UGCG^OE
4: BT-474_ZFX^OE

S5 Fig

C

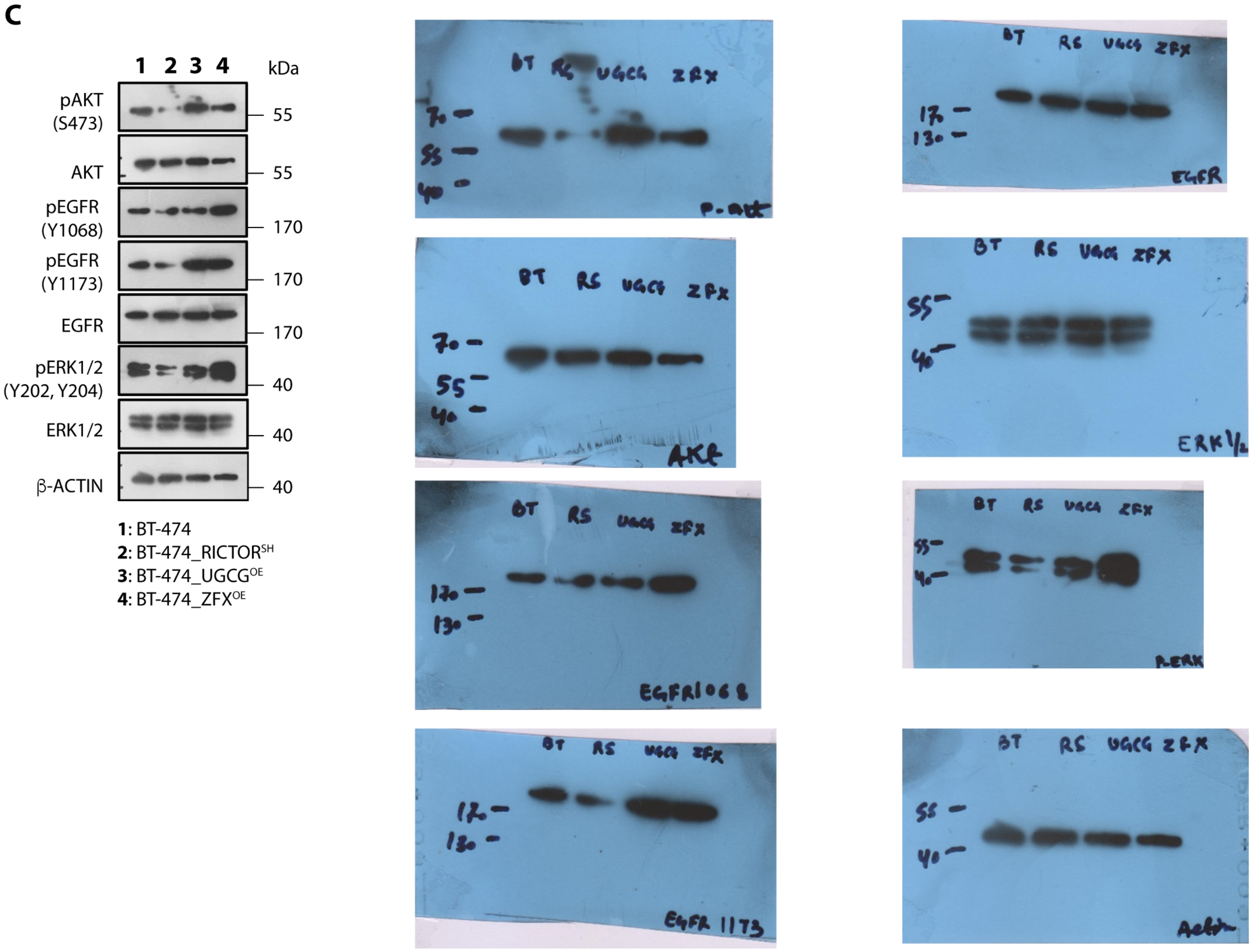
1 2 3 4 kDa
pAKT (S473)
55
AKT
55
pEGFR (Y1068)
170
pEGFR (Y1173)
170
EGFR
170
pERK1/2 (Y202, Y204)
40
ERK1/2
40
β-ACTIN
40
1: BT-474
2: BT-474_RICTOR^SH
3: BT-474_UGCG^OE
4: BT-474_ZFX^OE

D

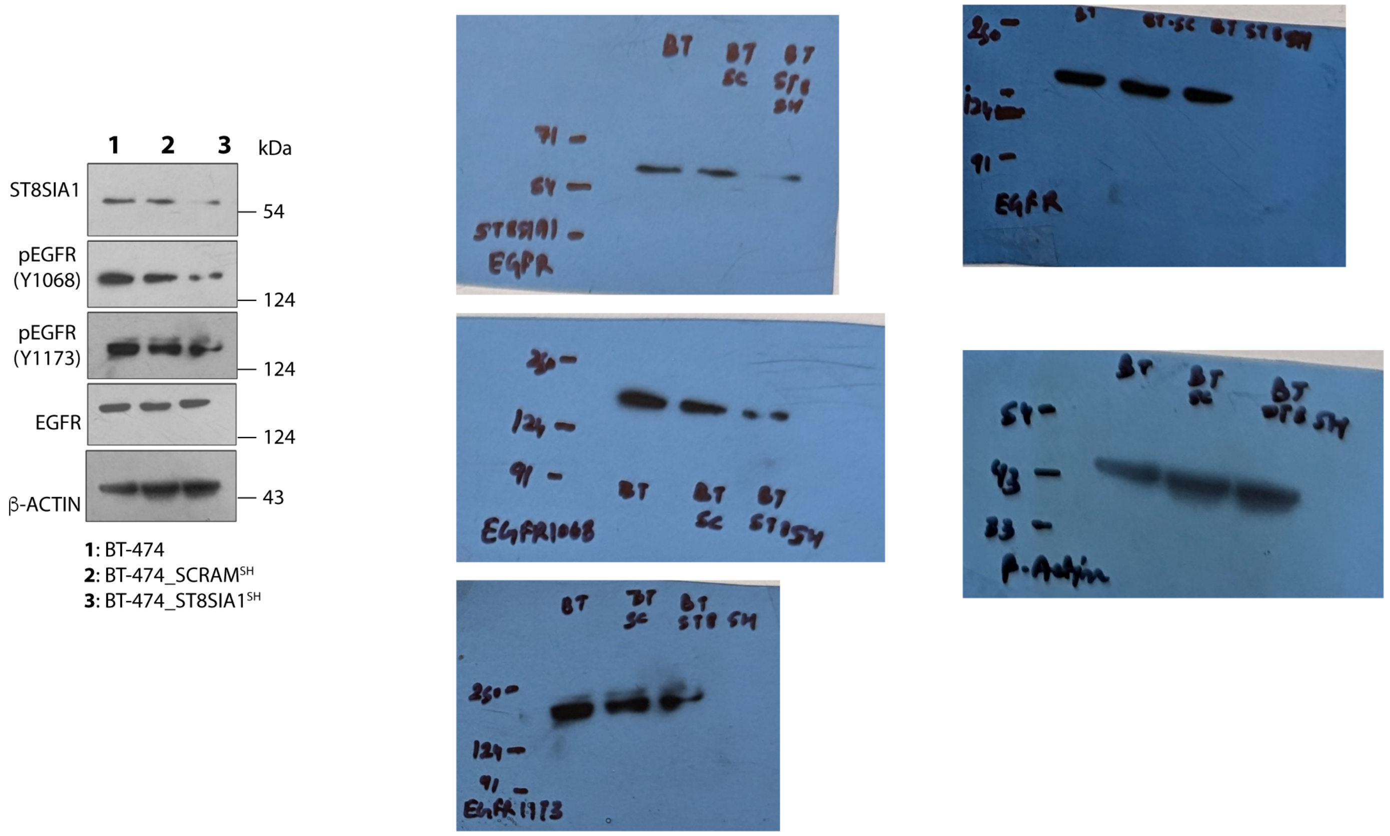
1 2 3 kDa
ST8SIA1
54
pEGFR (Y1068)
124
pEGFR (Y1173)
124
EGFR
124
β-ACTIN
43
1: BT-474
2: BT-474_SCRAM^SH
3: BT-474_ST8SIA1^SH

S5 Fig

F

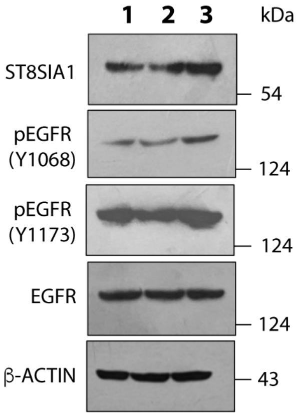

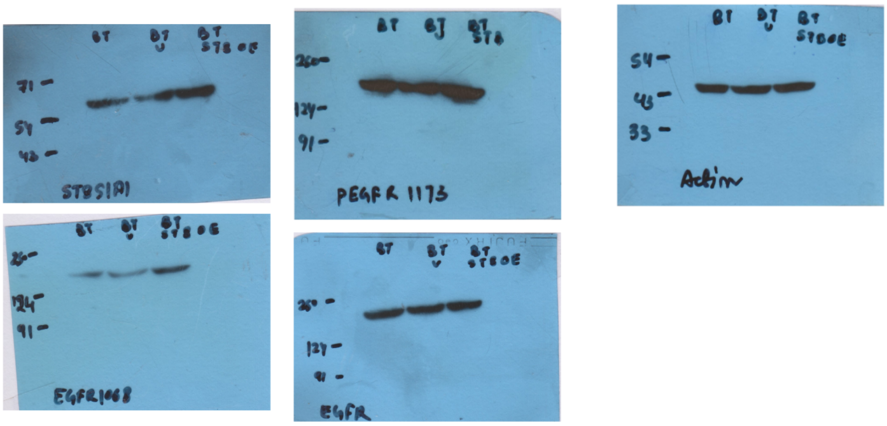

1: BT-474
2: BT-474_VECT^{OE}
3: BT-474_ST8SIA1^{OE}

I

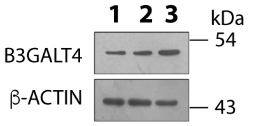

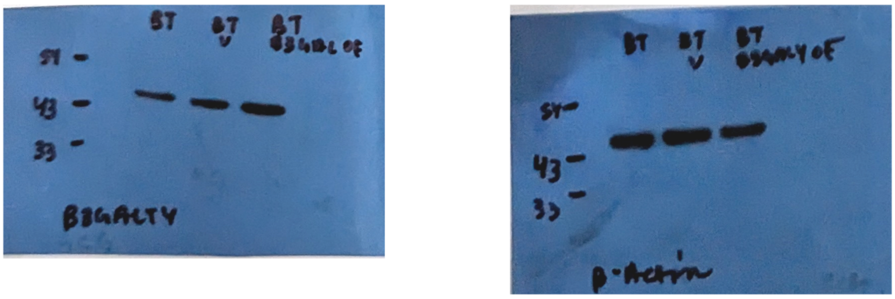

1: MCF-7
2: MCF-7_VECT^{OE}
3: MCF-7_B3GALT4^{OE}

M

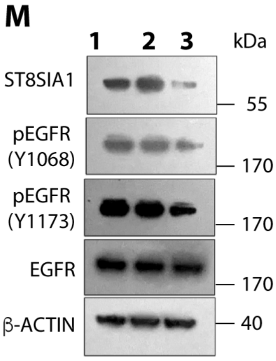

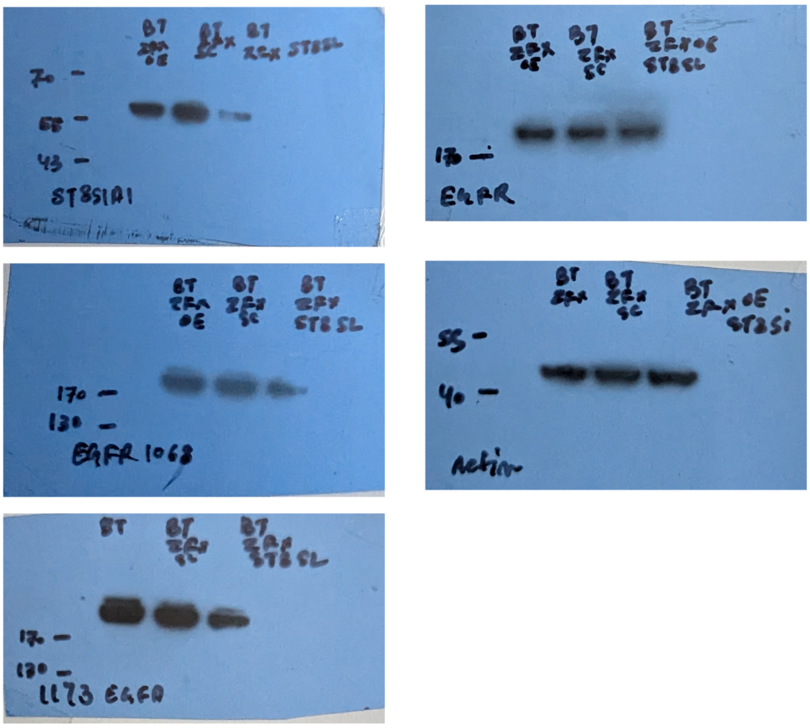

1: BT-474_ZFX^{OE}
2: BT-474_ZFX^{OE} + SCRAM^{SL}
3: BT-474_ZFX^{OE} + ST8SIA1^{SL}
