# Supplementary material for: The mTORC2 subunit RICTOR drives breast cancer progression by promoting ganglioside biosynthesis through transcriptional and epigenetic mechanisms
Source: PLoS Biol. 2025 Sep 11;23(9):e3003362. doi: 10.1371/journal.pbio.3003362 (PMC12425323; doi:10.1371/journal.pbio.3003362)
Supplement: Attachment — Submitted filename: Response to Reviewers.pdf [file pbio.3003362.s025.pdf]

Response to Reviewer's Comments

Reviewer 1

Comment 1. One of the surprising findings is how overexpression of UGCG and ZFX results in a decrease of GM1. The authors show no difference in the enzymes for GSL synthesis but do not explore whether there are changes in enzymes for breaking down complex GSL. Evaluating expressions of HEXA/B, GLA/GLB would help elucidate this difference. Additionally, showing the changes to the GSL profile upon ST8SIA1 siRNA treatment would provide a more complete picture.

Response. As suggested by the reviewer, we have checked the expression of neuraminidases (NEU1-4), hexaminidases (A/B) and GLA/GLB on overexpression of UGCG and ZFX as well as on RICTOR silencing in MCF-7 and BT-474 cells. Please see **Figure S5A and Supplementary S5B.**

As suggested by the reviewer, we also quantified the changes in gangliosides on overexpression and silencing of ST8SIA1 in MCF-7 and BT-474 cells. As we observed the significant changes only in GD3 gangliosides, we have added these results in **Figure 5F and Supplementary S5H.**

Comment 2. The authors do not show GM2/GD2 profiles throughout the paper; however these have also been shown to promote tumor growth and RTK signaling. While the impact of UGCG OE and increased GSLs on luminal breast cancer progression is clear, to show the mechanistic impact of GSLS, as the authors claim, it would be beneficial to include all major gangliosides in the pathway.

Response. As suggested by the reviewer, we have thoroughly revised the manuscript and included the quantification of all major gangliosides (GM3, GD3, GD2, GM2, GM1) in the pathway under different conditions.

Comment 3. The experiment performing the addition of GM1 and GD3 is not clear. In the methods, the authors describe dissolving both GSLs in serum free media but not how the cells were cultured. If the experiments were performed in serum free media, the elimination of growth factors and other components present in serum make it difficult to establish the impact solely of extracellular GSLs. While adding exogenous GSLs is not the same as those properly trafficked and synthesized endogenously, the authors should at least use delipidated FBS to maintain serum components. If serum was used in the media, methods need to be revised. Using an OE system for ST8SIA1 and B3GALT4 would be a cleaner way to increase flux towards those GSLs and capture the impact of endogenous synthesis.

Response. We thank the reviewer for these suggestions.

As suggested by the reviewer, we re-performed feeding experiments with glucosylceramides and gangliosides in media with lipid-free serum. We also used vehicle control in our experiments, and the data was normalised to vehicle control. Please see **Figure 5J and Supplementary Figure S5L.**

We also overexpressed and silenced ST8SIA1 in MCF-7 and BT-474 cells to alter the flux towards GD3 gangliosides and to capture the impact of endogenous synthesis of GD3 gangliosides on cell proliferation. Please see **Figure 5B-5F and Supplementary Figure S5D-S5H.**

We also overexpressed B3GALT4 in MCF-7 and BT-474 cells to increase the flux towards GM1 gangliosides and showed the impact of any alteration in endogenous synthesis of GM1 gangliosides on cell proliferation. Please see **Figure 5G-5I and Supplementary Figure S5I-S5J.**

Minor points:

Comment 4. The authors use the ratio of glucosylceramides to their respective ceramides to show that there is greater UGCG expression. It would be good to demonstrate that GSLs downstream of glucosylceramide are also increased, since the GSLs with greatest impact on signaling and RTK activity are downstream of UGCG activity.

Response. As suggested by the reviewer, we quantified the gangliosides in patient samples and included them in the revised manuscript. Please see **Figure 1C-1H.**

Comment 5. In Figure 2O, the authors should include the lipidomic profile or comparison to show if UGCG OE with ZFXSL impacts the GSLs in the cell even with a rescue.

Response. As suggested by the reviewer, we quantified the impact of UGCG_OE with ZFX silencing on gangliosides. Please see **Supplementary Figure S3T** in the revised manuscript.

Comment 6. In figure 3R, the authors show how ZFXOE + UGCGSL exhibits reduced growth as compared to just ZFXOE, but a comparison with a UGCGSL would benefit to deconvolute the impact of ZFXOE to other binding sites that may promote growth.

Response. As suggested by the reviewer, we compared the proliferation rate of ZFXOE + UGCGSL with that of UGCGSL in MCF-7 and BT-474 cells. Please see **Figure 3U and Supplementary Figure S3R.** We also included the comparison of the proliferation of ZFX-SL + UGCG-OE with UGCG-OE cells. Please see **Figure 3V and Supplementary Figure S3S.**

Comment 7. Some of the fold changes presented don't seem to match those of the figures. Fig 5B for example does not match the 4-fold described in the text.

Response. As suggested by the reviewer, we have corrected the fold changes throughout the manuscript.

Comment 8. The authors focus on ganglioside metabolism, but neutral glycosphingolipids will also be altered because of UGCG overexpression. Showing how neutral GSLs that will impact membrane dynamics and cell-cell contacts will aid in generating a mechanistic picture.

Response. As suggested by the reviewer, we quantified the changes in neutral glycosphingolipids (Globosides like Gb3, Gb4, Gb5) on UGCG overexpression in MCF-7 and BT-474 cells as shown below. We observed increased Gb4 globosides on UGCG overexpression in MCF-7 cells, whereas Gb3 globosides were significantly upregulated and Gb4 globosides downregulated in BT-474 cells on UGCG overexpression. These changes in globosides may alter the membrane dynamics and cell-cell contact, but in this manuscript, we would like to focus on the RICTOR-mediated regulation of gangliosides.

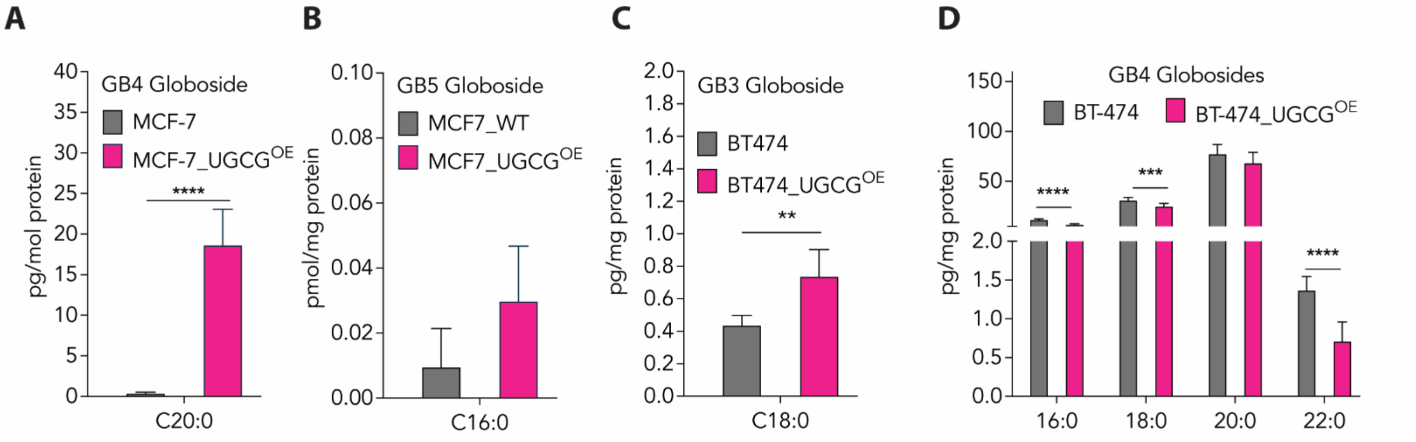

Comment 9. One of the limitations in performing the experiments in NOD mice is losing the immune interactions that will be altered as cells present an increase of GSLs, altering their interactions with other tumor cells, extracellular matrix, and the immune system. This should be discussed as a limitation, or some work could be done to elucidate how changes in GSL profile affect growth.

Response. We completely agree with the reviewer about the limitation of performing experiments in NOD mice. We have discussed this limitation in the revised manuscript. We want to add that additionally in our lab, we are actively pursuing a project on deciphering the impact of GSLs on the tumour microenvironment in syngeneic murine models, however, that is not in the scope of this manuscript.

Comment 10. No methods are shown for BT474 flank injections.

Response. We have included the methods for all animal experiments in the revised manuscript.

Comment 11. A discussion about why cells were not injected into mammary fat pads would be good as this would be more representative of the local environment.

Response. We want to state that as the animal experiments were done on 4-6-weeks-old female virgin NOD mice, these mice do not have a well-developed mammary fat pad. As suggested by the reviewer, we repeated experiments by injecting cells in the mammary fat pad and did not find any significant differences in our results.

Comment 12. The authors focused on luminal breast cancer lines due to TCGA dataset results. However, they also tested siRNA on HCT116s, HEPG2s, and MDA-MB-453s (TNBC) and saw a similar reduction in UGCG expression upon ZFXSL. Some discussion or results suggesting whether this response of increased growth because of RICTOR-ZFX-UGCG axis is only present in luminal breast cancer, or other lines would help to highlight the impact of these results for combination therapies and strategies as the authors mention in the discussion.

Response. As suggested by the reviewer, we have included the importance of the RICTOR-ZFX-UGCG axis on cancer cell proliferation, with discussion on whether it is only restricted to a luminal subtype of breast cancer or can be observed in other cell lines.

Reviewer 2

Comment 1. The observation that RICTOR regulates glycolipid metabolism is interesting and important. However, authors show this observation only in 2 breast cancer cell lines. It would be interesting to at least demonstrate this key finding in a broader range of cells (including non-diseased cells).

Response. As suggested by the reviewer, we have quantified the changes in glycolipids in HCT-116 (colon cancer cell line) and HEK-293 (normal transformed cell line). Please see the **Supplementary Figure S2N-S2Q**.

We want to emphasise that our bioinformatic study identified three transcription factors, ZFX, CTCF, and ELF1, to be transcriptionally regulated by RICTOR in MCF-7 and BT-474 cells. These transcription factors may be regulating many other genes, including some genes of sphingolipid and ganglioside pathways, in a cell-type and cancer-context-dependent manner. Their regulatory mechanisms and signalling targets may also vary in different cancer types. Deciphering all of this, however, is beyond the scope of the study, as the focus of this manuscript is on luminal breast cancer. Therefore, we have performed and validated all our results in two luminal breast cancer cell lines, MCF-7 and BT-474.

Comment 2. Moreover, authors use the BT-474 cell line to confirm their findings in MCF-7 cells, but only include selected experiments and do not confirm multiple key experiments in the second cell line.

Response. As recommended by the reviewer, we have now included all the experiments in the MCF-7 and BT-474 cell lines and revised the manuscript extensively.

Comment 3. Regarding the in vivo experiment with Eliglustat: the method of administration is not an accepted method. Also the single dose is low (25 mg/kg), and lacks biomarker confirmation. Moreover, the reference (ref 27) given to the details of the method does not contain further details of the method. Finally, the control arm for this experiment was "untreated" animals - lacking vehicle control. Taken together, these points severely limit any conclusion from Fig. 6L-O.

Response. As suggested by the reviewer, we have repeated the animal experiments upon intraperitoneal injection of Eliglustat (60 mg/kg/daily) and used a vehicle as a control. These results are revised in **Figure 6L, 6N**.

We also quantified and confirmed the changes in glucosylceramides and gangliosides in tumour tissues upon eliglustat treatment. These results are included in **Figure 6M, 6O, Supplementary Figure S6J, S6K**.

Comment 4. The major limitation of this study is the substantial lack of experiments with supplementation of glucosylceramide as well as more complex glycolipids such as gangliosides. The authors only include 1 example of such phenocopy/rescue experiment where GD3 or GM1 ganglioside is supplemented to RICTOR KD or UGCG OE MCF-7 cells, respectively (Fig. 5N ,O). However, the use of untreated control instead of vehicle control, as well as the double normalization (untreated condition for all cells set to 100%) limits conclusive interpretation of these data. In general, the absence of such data prevents any solid conclusion on the molecular mechanism that the authors wanted to explore in this manuscript.

Response. We thank the reviewer for these suggestions.

As suggested by the reviewer, we re-performed feeding experiments of glucosylceramides and gangliosides in media with lipid-free serum. We have used vehicle control in our experiments, and normalised the data to vehicle control. Please see **Figure 5J and Supplementary Figure S5L.**

We overexpressed and silenced ST8SIA1 in MCF-7 and BT-474 cells to alter the flux towards GD3 gangliosides and to capture the impact of endogenous synthesis of GD3 gangliosides on cell proliferation. Please see **Figure 5B-5F and Supplementary Figure S5D-S5H.**

We also overexpressed B3GALT4 in MCF-7 and BT-474 cells to increase the flux towards GM1 gangliosides and showed the impact of any alteration in endogenous synthesis of GM1 gangliosides on cell proliferation. Please see **Figure 5G-5I and Supplementary Figure S5I-S5J.**

Minor comments

Comment 5. The authors show elevated expression of RICTOR, ZFX, and UGCG in bulk RNA data from patients with cancer to suggest elevated expression in cancer cells in situ. However, it is important to verify cancer cell specific overexpression by analysis of publicly available or proprietary single cell RNA data.

Response. We analysed the expression of RICTOR, ZFX, and UGCG from publicly available single-cell RNA data and compared the expression of RICTOR, ZFX, and UGCG in cancer cells and stromal cells. These results validated cancer cell-specific expression of RICTOR, ZFX, and UGCG, which is included in the revised manuscript. Please see **Supplementary Figure S6I-S6J.**

Comment 6. Authors use UGCGDEAD as a control in several experiments, but fail to include a confirmation experiment that demonstrates the inactive state of this gene product that they overexpress. This could be done by lipidomics.

Response. As suggested by the reviewer, we have added the quantification of glucosylceramides from UGCG-DEAD cells in the revised manuscript. Please see **Supplementary Figure S1O, S1P**.

Comment 7. Lipidomics data from patients, where selected ceramide and glucosylceramide species are compared between tumor and adjacent normal tissues are now represented and statistically analyzed in an unpaired manner. However, given these samples are "matched" (from the same patients), they should be analyzed as such with a paired statistical test and this could have major implications on the observed effect and associated conclusion.

Response. As suggested by the reviewer, we performed a paired statistical analysis and revised the Figures. Please see revised **Figure 1B-1H, S1A, S1B**.
