# Supplementary material for: The mTORC2 subunit RICTOR drives breast cancer progression by promoting ganglioside biosynthesis through transcriptional and epigenetic mechanisms
Source: PLoS Biol. 2025 Sep 11;23(9):e3003362. doi: 10.1371/journal.pbio.3003362 (PMC12425323; doi:10.1371/journal.pbio.3003362)
Supplement: Attachment — Submitted filename: 2_Response to Reviewers.pdf [file pbio.3003362.s026.pdf]

## Response to Reviewer's Comments

**Reviewer 2.**

The authors have performed a substantial amount of additional work that has significantly improved the quality of the manuscript.

**Major comment:** Figure 6O shows increased abundance of glucosylceramides in BT-474 tumours upon Eliglustat treatment, in contrast to Figure 6L and the expected effects of UGCGi. The manuscript currently lacks in a description and a discussion for this finding.

**Response.** We have included the discussion on these findings in the revised manuscript.

**Minor comments:** Overall, the manuscript would benefit from more precise phrasing when reporting increases or decreases, as it often omits the specific baseline or comparator to which these changes refer. It also lacks essential details on the timing of experiments, such as the duration of cell or mouse treatments prior to measurements, which is critical for the reproducibility of results.

**Response.** As suggested by the reviewer, we have revised the manuscript with precise phrasing. All the details regarding the timing of experiments, concentration of drugs used and the respective duration of cell or mouse treatments have been mentioned in the methods section.
